# Multi-modal data to identify key factors influencing lung injury in ARDS patients undergoing invasive mechanical ventilation: A prospective multi-center observational study protocol

**DOI:** 10.1371/journal.pone.0332985

**Published:** 2026-01-23

**Authors:** Zhimei Duan, Di Lian, Kaifei Wang, Ye Hu, Han Fu, Ruoxuan Wen, Ying Zhao, Xingshuo Hu, Pan Pan, Jianqiao Xu, Jin Chen, Li Xiao, Lin Wang, Xiao Yu, Xiaobo Han, Wuxiang Xie, Fei Xie, Lixin Xie, Zhihai Han

**Affiliations:** 1 College of Pulmonary and Critical Care Medicine, Chinese PLA General Hospital, Beijing, China; 2 Naval Clinical College of Anhui Medical University, Beijing, China; 3 The Fifth Clinical Medical College of Anhui Medical University, Hefei, China; 4 Peking University Clinical Research Institute, Peking University Health Science Center, Beijing, China; Children's National Hospital, George Washington University, UNITED STATES OF AMERICA

## Abstract

**Background:**

Patients with moderate to severe acute respiratory distress syndrome (ARDS) exhibit extremely poor prognoses following mechanical ventilation, with mortality rates as high as 40% to 55%. Despite extensive research into ARDS classification and prognostic assessment, the disease’s pathogenesis remains incompletely understood, and there remains a critical lack of specific biomarkers and effective therapeutic targets for its prevention and management. The core challenges lie in two key areas. First, ARDS demonstrates marked heterogeneity in etiology, pathophysiology, and pathogenesis. Existing research, predominantly reliant on population-level average data, fails to capture inter-individual variability, hindering the precise identification of patient subgroups responsive to specific therapeutic regimens. Second, current definitions of ARDS phenotypes are often confined to clinical symptoms and routine diagnostic indices, lacking integrated analysis of deeper mechanistic indicators, such as key biomarkers and respiratory mechanics parameters, thereby limiting the stability and clinical utility of existing classification systems.

**Methods/Design:**

We designed a prospective multicenter cohort study incorporating multi-omics analyses. This research aims to investigate the mechanisms underlying the development and progression of ARDS during mechanical ventilation, providing a theoretical foundation and practical guidance for future ARDS therapies. The study plans to enroll over 165 patients with moderate to severe ARDS receiving mechanical ventilation across 10 medical centers. Peripheral blood and bronchoalveolar lavage fluid (BALF) samples will be collected on the first 24 hours after enrollment and at extubation for metagenomic/meta-transcriptomic sequencing, bulk RNA sequencing, single-cell RNA sequencing, proteomics detection, and metabolomics analyses. Concurrently, comprehensive monitoring of physiological indices, electrical impedance tomography, transpulmonary pressure, pulmonary ultrasound findings, and other relevant parameters will be conducted during the enrollment. Study participants will be stratified by survival and mortality outcomes to analyze the dynamic trends of all measured indices and their underlying molecular mechanisms. Biomarkers derived from multi-omics data and clinical baseline characteristics will be evaluated and integrated, followed by multidimensional dimensionality reduction. Predictive models will be subsequently constructed via early or late fusion to identify core prognostic markers, with performance validated using standardized metrics.

**Discussion:**

Through comparative analysis of multi-omics data, we aim to identify specific markers and risk factors associated with distinct clinical trajectories of ARDS, further clarifying the key determinants of lung injury. Ultimately, this research will reveal critical immune cell subtypes that govern ARDS onset and prognosis, offering novel insights and therapeutic targets to advance precision medicine for ARDS.

**Study protocol registration:**

ClinicalTrials.gov NCT05922826.

## Introduction

Acute Respiratory Distress Syndrome (ARDS) is a complex clinical syndrome triggered by various risk factors such as pneumonia, infection, trauma, blood transfusion, burns, aspiration, and shock [1,2]. Its core pathological change is increased permeability of the pulmonary vascular endothelium and alveolar epithelium, leading to pulmonary edema and gravity-dependent atelectasis. Characterized by bilateral pulmonary infiltrates and non-cardiogenic pulmonary edema, ARDS presents with progressive respiratory distress and refractory arterial hypoxia [3–6]. Due to the high clinical and biological heterogeneity among patients, there is currently no radical treatment and the global in-hospital mortality rates remaining as high as 40%−55% worldwide [7,8]. Currently proven effective interventions are limited to supportive therapies such as lung-protective mechanical ventilation, prone position ventilation and high positive end-expiratory pressure (PEEP).

Research related to mechanical ventilation has long been the focus of ARDS clinical research. Electrical impedance tomography (EIT) can evaluate the degree of pulmonary collapse in gravity-dependent areas of patients with COVID-19 secondary to ARDS, and the degree of collapse in these areas in the supine position is significantly associated with the improvements in oxygenation during prone positioning [9]. Transpulmonary pressure guided individualized PEEP titration can reduce lung injury and improve prognosis [10]. Critical care ultrasound can real-time monitor ventilation changes during the lung recruitment process through the Lung Ultrasound Score (LUS), facilitating the individualized titration of mechanical ventilation parameters and prognostic evaluation [11–13].

In recent years, the rapid development of molecular biology and bioinformatics has gradually deepened our understanding of the pathophysiology of ARDS. Addressing disease heterogeneity, researchers have used multi-omics technologies such as genomics, transcriptomics, proteomics, metabolomics and metagenomics, to identify subtype-specific therapeutic targets [14–17]. A study on pediatric ARDS identified three transcriptome subtypes (CATS) through cluster analysis of whole blood microarrays, which are respectively associated with pathways related adaptive immunity, complement and G protein-coupled receptor signaling. The mortality of the CATS1 subtype (32%) is significantly higher than that of CATS3 subtype (8%) [18], providing a basis for clinical precise diagnosis and treatment.

Studies on molecular markers have shown that high expression of genes such as *ELAVL-1/HUR* and *GSK3β* is related to the poor therapeutic outcomes in ARDS [19], and high expression levels of p300/CBP in peripheral blood is a risk factor for 28-day mortality [20]. Single nucleotide polymorphisms (SNP) of genes such as *TNF-α* (*rs1800629)*, *IL-6 (rs1800796)*, and *MyD88 (rs7744)* are closely related to the risk of onset and prognosis [21]. The gene expression heterogeneity of alveolar macrophages (AMs) and Peripheral Blood Mononuclear Cells (PBMCs) is significant, and high expression of pro-inflammatory genes in PBMCs often indicates poor outcomes [22]. Down-regulation of genes such as *METTL16* and *FTO* participates in inflammation and lung injury progression by regulating lipopolysaccharide-induced m6A methylation [23].

Proteomic and metabolomic studies have also revealed the characteristics of ARDS subtypes. Analysis of bronchoalveolar lavage fluid (BALF) from ARDS patients shows that the activation of immune response, wound healing, and multiple pathways is more significant in non-survivors [24]. The levels of metabolites such as isoleucine and leucine in BALF, and proline in serum, combined with association with Chronic Health Evaluation (APACHE) II and Sequential Organ Failure Assessment (SOFA) scores, can effectively predict mortality [24,25]. Metagenomic studies have found that changes in the diversity of the lower respiratory tract microecology in ARDS patients are closely related to disease severity and prognosis [26,27], and whole-exome sequencing has identified potential ARDS-related SNP markers [28].

Despite extensive research on ARDS typing, accurate treatment and poor prognosis, the disease progression of ARDS remains unclear, and there are still no specific biomarkers and effective targets for prevention and treatment for ARDS. In the era of precision medicine, integrating multi-demensional information including clinical features, imaging, physiology, biological tests, and multi-omics analyses) to achieve accurate ARDS subtyping is a core research direction for realizing “treatment-feature-matching” and improving the accuracy of therapeutic effect prediction [2,7,8]. This study protocol intends to collect clinical indicators, ventilation parameters, lung ultrasound, and EIT data from multi-center mechanically ventilated patients with moderate to severe ARDS, and integrate multi-omics data including metagenomics, bulked and single-cell transcriptomics, proteomics and metabolomics data from their blood and BALF samples. By summarizing changes in respiratory therapy-related parameters and exploring quantitative indicators for evaluating lung injury, the study protocol aims to identify key factors affecting the occurrence, development, and prognosis of ARDS, providing a basis for precise treatment of the disease.

## Materials and methods

### Purpose of the study

The study aims to identify key risk factors associated with differential survival and mortality outcomes in mechanically ventilated patients with moderate or severe ARDS who exhibit varying prognoses. This objective will be accomplished by investigating the differences and dynamic changes in clinical examinations, respiratory mechanics, lung ultrasound findings, and multi-omics analyses, as well as by further exploring their associations with prognosis. Additionally, the study aims to screen for functionally significant genes and candidate pathways, with a specific focus on lung injury-related mechanistic pathways, and identify the key immune cell subtypes (e.g., alveolar macrophages, T cell subsets, B cells, and myeloid cells) that determine the onset and prognosis of ARDS. Collectively, these efforts aim to provide novel insights and therapeutic targets to advance the precision treatment of ARDS.

### Study design

**Fig 1:**
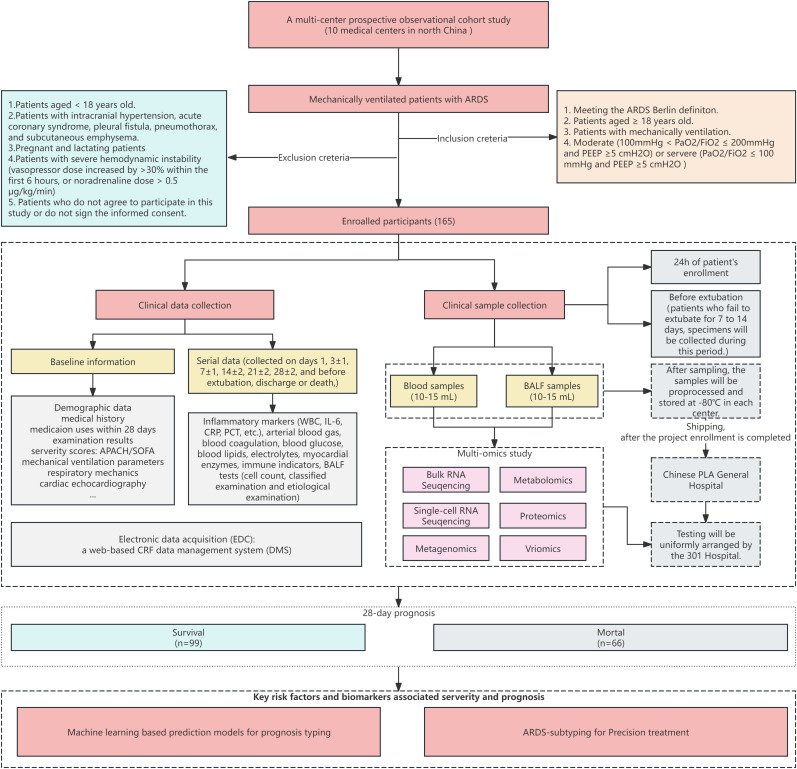
Flowchart showing the study design. Eligible patient meets the inclusion and exclusion criteria and written informed consent will be enrolled. Baseline clinical data and biological samples will be collected immediately upon enrollment. Blood and BALF samples will be obtained on the first day of enrollment and prior to extubation. Throughout the study period, comprehensive clinical data, including laboratory test results, monitoring parameters, and treatment records, will be prospectively gathered. The 28-day clinical outcomes will also be documented. The collected samples will undergo multi-omics analyses, specifically transcriptomics, proteomics, metabolomics, and genomics. Clinical data will be further scrutinized using stratification analysis. Finally, all data will be integrated, and machine learning approaches will be employed to identify and characterize the key factors contributing to lung injury in ARDS patients undergoing invasive mechanical ventilation.

**Fig 2:**
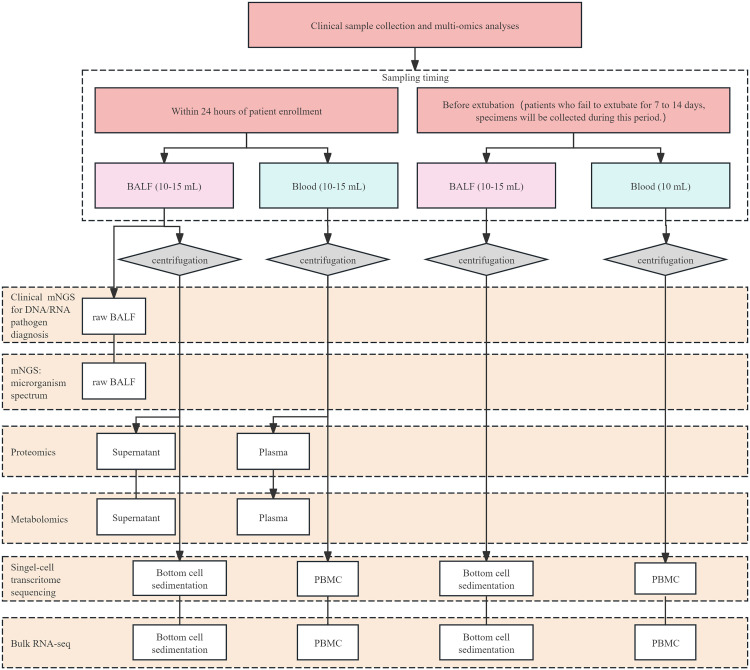
Sample collection, pretreatment, and multi-omics analysis pipeline. A total of six multi-omics assays will be conducted, with sample utilization stratified by type and collection time point. Bronchoalveolar lavage fluid (BALF) samples collected within 24 hours of enrollment will first utilize the original fluid for clinical mNGS for pathogen diagnosis, alongside metagenomic microecology analysis and virus spectrum analysis. All samples will undergo centrifugation within 24 hours of collection. Following centrifugation, the BALF supernatant will be subjected to proteomic and metabolomic assays, while the resulting cell pellet will be utilized for transcriptomic and single-cell transcriptomic analyses. Concurrently collected blood samples will also be centrifuged, with the plasma used for proteomic and metabolomic assays, and peripheral blood mononuclear cells (PBMCs) utilized for bulk transcriptomic and single-cell transcriptomic analyses. For the late phase, BALF and blood samples collected prior to extubation (with priority given to day 10 ± 2) will focus on transcriptomic and single-cell transcriptomic analyses: centrifugation-derived BALF cell pellets and blood PBMCs will be exclusively used for these assays.

In this study, a prospective multicenter clinical cohort study will be conducted to observe the changes of lung injury, EIT, transpulmonary pressure, lung ultrasound, and other indexes in patients with ARDS. The effects on lung injury in patients with ARDS from the perspectives of respiratory mechanics, ventilation and perfusion, and lung imaging will be evaluated. The key indicators to provide clinical data support for the establishment of a 28-day death prediction model for patients with ARDS will be screened. The detailed technical route is shown in the following Fig 1. The specific sampling strategies and multi-omics detections were illustrated in Fig 2.

### Participant recruitment

Participants will be recruited from ten medical centers in tertiary general hospitals in North China, including the First Medical Center of Chinese PLA General Hospital, the Sixth Medical Center of Chinese PLA General Hospital, the Eighth Medical Center of Chinese PLA General Hospital, Beijing Shijitan Hospital, Beijing Anzhen Hospital, AMHT Group Aerospace 731 Hospital, Zhengzhou Central Hospital, Henan Provincial People’s Hospital, the First Affiliated Hospital of Zhengzhou University and First Hospital of Shanxi Medical University.

Patients with ARDS will be invited to participate in this study upon admitting the branch center. Those who meet the inclusion criteria and provide signed informed consent will enter the screening period (S1 File). During the screening visit, trained researchers (physicians) will confirm the eligibility based on symptoms, laboratory results, and chest X-ray or CT image, and review every inclusion and exclusion criterias. Patients who meet the exclusion criteria will be excluded from this study within 24 hours. The recruitment period is set for 24 months, spanning from November 2023 to November 2025.

### Inclusion and exclusion criteria

In this study protocol, patients with moderate or severe ARDS and experienced mechanical ventilation will be included for inclusion selection. The definition of ARDS refers to the Berlin definition of ARDS [3]. The core diagnostic criteria include: an onset time within 1 week after a known clinical insult or the onset of new/worsening respiratory symptoms; chest imaging findings of bilateral lung opacity or patchy shadows that cannot be fully explained by conditions such as pleural effusion, atelectasis, or nodules; respiratory failure that cannot be fully attributed to heart failure or fluid overload, with objective examinations such as echocardiography required to rule out hydrostatic pulmonary edema in the absence of clear predisposing risk factors; and disease severity classified into three grades based on the oxygenation index (PaO₂/FiO₂) combined with a positive end-expiratory pressure (PEEP ≥ 5 cmH₂O): mild (200 mmHg < PaO₂/FiO₂ ≤ 300 mmHg, with continuous positive airway pressure ventilation permitted), moderate (100 mmHg < PaO₂/FiO₂ ≤ 200 mmHg), and severe (PaO₂/FiO₂ ≤ 100 mmHg). Patients with moderate or severe ARDS will be included.

Patients meeting any of the following criteria will be excluded from the study:

1. Patients aged less than 18 years old.
2. Patients with intracranial hypertension, acute coronary syndrome, pleural fistula, pneumothorax, and subcutaneous emphysema.
3. Pregnant and lactating patients.
4. Patients with severe hemodynamic instability (vasopressor dose increased by >30% within the first 6 hours, or noradrenaline dose > 0.5 μg/kg/min).
5. Patients who do not agree to participate in this study and patients do not sign the informed consent.

### Data collection

The formal baseline data and serial data of the participants will be collected. Three case report forms (CRFs) have been designed for different stages of patient enrollment management (S2–S4 Files).

The formal baseline data includes the following aspects:

Demographic data: hospitalization number, name, sex, age, main cause of admission, ethnicity, smoking history, and body mass index.

Medical history: hypertension, coronary heart disease, diabetes, kidney disease, hepatopathy, cerebrovascular disease, autoimmunity disease, hemopathy, pulmonary disease, and cancer.

Medication use within 28 days: antibiotics, hormones and immunosuppressants, vasoactive agent, neuromuscular blockers, sedative and analgesic drugs.

Physical examination findings: blood pressure, pulse rate, heart rate, respiratory rate, and temperature.

Severity scores: the Acute Physiology and APACHE II and the SOFA score.

Mechanical ventilation parameters (on day 1 and before extubation): ventilation mode, inhaled oxygen fraction, PEEP, frequency, tidal volume, flow rate, expiratory tidal volume, peak pressure, plateau pressure, airway resistance.

Respiratory mechanics (on day 1 and before extubation): the respiratory system compliance (C_rs_), static compliance of the lungs (C_lung_), respiratory system airway resistance (R_rs_), esophageal pressure (P_es_), transpulmonary pressure (P_tp_), transpulmonary driving pressure (△P_tp_), driving pressure (△P), dead space fraction (V_D_/V_t_) and mechanical power (MP).

Cardiac echocardiography (on day 1 and before extubation): measurement of left ventricular ejection fraction.

Pulmonary ultrasound score and pulse indicator continuous cardiac output parameters.

Serial data: The serial data will be collected on days 1, 3 ± 1, 7 ± 1, 14 ± 2, 21 ± 2, 28 ± 2, and before extubation, discharge or death, including inflammatory markers (white blood cell count, neutrophil count and proportion, interleukin-6, C-reactive protein, procalcitonin, etc.), arterial blood gas, coagulation, blood glucose, blood lipids, electrolytes, myocardial enzymes, immune indicators, BALF tests (cell count, classified examination and etiological examination).

### Data management

In this protocol, we employ a web-based CRF data management system (DMS) for data collection and centralized management throughout the experiment. The DMS records patients’ enrollment details, clinical treatment data, and sample collection information (e.g., time, location, handling procedures), enabling full-lifecycle patient data management, including screening and real-time monitoring of adherence to predefined inclusion/exclusion criteria. After new participants are enrolled, data collectors will visit sub-centers weekly to input required information into the online DMS (https://h6world.cn). The data administrator is responsible for collecting data monitored by independent external supervisors. Questions or outliers should be answered by the quality control officer. 10% of the subjects will be randomly selected and tested with the original questionnaire to evaluate the bit error rate. Based on the bit error rate, the subsequent monitoring plan will be arranged. If an error occurs, the incorrect data in the database must be corrected and all modifications must be documented in the DMS. Once data entry and resolution of issues are complete, the research data will be locked by the lead researcher, preventing any further alterations by the researchers. No compensation will be provided.

### Sample collection, and multi-omics profiling

For enrolled patients, peripheral blood and BALF samples will be collected at two time points: within 24h after enrollment and before tracheal extubation. For patients who cannot be extubated within 7–14 days, samples will be collected during this 7–14 day window, with priority given to day 10 ± 2 (Fig 2).

For blood sampling, patients must be in a fasting state prior to blood collection. A total of 10–15 mL of blood will be collected using anticoagulant tubes, which will then undergo pretreatment before separate storage or detection. Within four hours of sampling, centrifugation will be conducted at 3000 r/min for 10 minutes. The resulting plasma will be aliquoted into 1.5 mL EP tubes at 500 μL per tube for subsequent proteomics and metabolites detection. For the remaining sample, equal volumes of phosphate buffered saline (PBS) and Ficoll lymphocyte separation solution will be added, followed by centrifugation at 1000g for 20 minutes to collect PBMCs. After adding 5 mL of PBS, the mixture will be centrifuged at 400g for 5 minutes, the supernatant will be discarded, and PBMCs will be resuspended in cell cryopreservation solution before aliquoting into EP tubes at 1 mL per tube. The PBMC samples will be used for RNA-seq and scRNA-seq. The samples will be stored at −80°C before multi-omics detection.

For BALF sampling, two tubes of BALF samples will be collected. One tube, containing no less than 10 mL, will undergo pretreatment for subsequent RNA-seq, scRNA-seq, proteomics and metabolites detection. The other tube, with a volume of at least 5 mL, will be directly submitted for clinical mNGS pathogen testing, as well as for metagenomic microbial spectrum analysis and meta-transcriptomic expression profiling. For reserved samples, 6 mL of BALF will be centrifuged at 4000g for 10 minutes. The supernatant will then be aliquoted into 1.5 mL EP tubes at 400 μL per tube, while 1 mL of Trizol will be added to the remaining cell pellet, followed by vortex mixing. All aforementioned samples, along with the remaining BALF, will be labeled and stored at −80°C. The supernatant will be used for proteomics and metabolites detection, while the bottom cell-sedimentation will be used for bulk RNA-seq and scRNA-seq. Since BALF sampling is invasive, the patient’s condition will be evaluated prior to sampling. If the attending physician determines that further sampling is inappropriate or the patient declines sampling, the procedure will not be performed. During sampling, certain adverse events will be documented. Subsequently, the attending physician will assess the feasibility of subsequent sampling. If the risk of further BALF sampling is deemed relatively high, the patient will be excluded from the study.

BALF samples collected for mNGS-based infectious disease diagnosis will be transported immediately to the corresponding testing center for mNGS pathogen detection. Any samples that could not be shipped on the day of collection should be temporarily stored at 2–8°C. Other collected samples will underwent appropriate preprocessing before being preserved at −80°C. Following the enrollment of all patients, all samples will be uniformly transported to the PLA General Hospital via cold chain logistics for multi-omics analysis. Crucially, all samples will be subjected to no more than one freeze-thaw cycle prior to testing to maintain sample integrity. The detailed methods for multi-omics detections are summarized in S5 File.

### Follow‑up schedules

At the 28-day mark, patients will be assessed for the primary outcome of survival or death. At this point, the attending physician will report the primary outcome, and the data collection database will then document the 28-day mortality rate, as well as the duration of ICU and hospital stays, along with other relevant outcomes. During the study period, specific measurements will be taken at predetermined time points: day 1, day 3 ± 1, day 7 ± 1, day 14 ± 2, day 21 ± 2, day 28 ± 2, and before extubation (with priority to day 10 ± 2), discharge or death. The following parameters will be recorded: inflammatory markers, arterial blood gas, coagulation, blood glucose, blood lipids, electrolytes, myocardial enzymes, immune indexes, cell count and classification of BALF and etiological examination. 28-day administration of antibiotics, neuromuscular blockers, vasoactive agent, hormones and immunosuppressants will be tracked. The measurement and collection schedule of this experiment are summarized in the follow-up form (Table 1).

**Table 1:** The schedule of measurements and visits of this trial.

Timeline (day)	Screening	Follow-up								
	d1	d3 ± 1	d7 ± 1	before extubation (priority: d10 ± 2)	d14 ± 2	d21 ± 2	d28 ± 2	discharged/death (≤28 days)	d1-28	d28
**Informed consent form**	Χ									
**Inclusion/exclusion criteria**	Χ									
**Demographic data**	Χ									
**History of diseases**	Χ									
**ARDS risk factors**	Χ									
**Clinical symptom**	Χ	Χ	Χ	Χ	Χ	Χ	Χ	Χ		
**Imaging**	Χ	Χ	Χ	Χ	Χ	Χ	Χ	Χ		
**APACHE Ⅱ scores**	Χ	Χ	Χ	Χ	Χ	Χ	Χ	Χ		
**SOFA scores**	Χ	Χ	Χ	Χ	Χ	Χ	Χ	Χ		
**mechanical ventilation parameter**	Χ	Χ	Χ	Χ						
**respiratory mechanics**	Χ			Χ						
**pulmonary ultrasound score**	Χ			Χ						
**cardiac echocardiography**	Χ			Χ						
**pulse indicator continuous cardiac output parameters**	Χ			Χ						
**mNGS detection**	Χ									
**Clinical pathogen detection**	Χ	Χ	Χ	Χ	Χ	Χ	Χ	Χ		
**Laboratory examination**	Χ	Χ	Χ	Χ	Χ	Χ	Χ	Χ		
**BALF cell count and classified examination**	Χ			Χ						
**Samples**	Χ			Χ						
**Antibiotic use record**									Χ	
**vasoactive agent use record**									Χ	
**neuromuscular blockers, sedative and analgesic drugs use record**									Χ	
**Adverse events**									Χ	
**28-day survival follow-up**										Χ
**28-day ICU length of stay**										Χ
**28-day ventilator-free days**										Χ

X indicates that the corresponding actions and clinical data will be performed or recorded during the specified time period. Abbreviations: d1: First day of enrollment; ARDS: Acute Respiratory Distress Syndrome; APACHE: Acute Physiology and Chronic Health Evaluation; SOFA: Sequential Organ Failure Assessmentm; mNGS: Metagenomic Next Generation Sequencing; BALF: Bronchoalveolar Lavage Fluid.

### Multi-Omics data analysis and integration

The core objective of day 1 metagenomic testing is to characterize the infection status of patients at the initial stage of intubation, including the presence of infection, pathogenic species, and strain types, thereby providing key references for subsequent patient stratification. Combined metagenomic and viromic analyses focus on unraveling microecological differences at baseline, exploring variations in microbial diversity and background flora composition across distinct patient groups. RNA-seq data aims to capture global differences in early host immune responses, ultimately identifying differentially expressed genes (DEGs) as well as enriched metabolic pathways and gene ontologies (GO terms). Single-cell transcriptomics enables more refined dissection of immune response patterns, clarifying heterogeneous differences in immune regulation among individual patients. Proteomics and metabolomics, respectively, investigate metabolism-specific and protein-specific differential molecules involved in the host immune processes of patients with divergent outcomes (survival vs. death), comprehensively covering functional molecular variations linked to immune regulation. While single-omics analyses can initially yield a large set of differential markers, subsequent multi-omics integration is essential to further prioritize and narrow down core candidate markers.

To accurately identify key regulatory factors, cross-omics integrated screening of proteomic, metabolomic, transcriptomic, and microecological data will first be performed, using gene function or metabolic pathways as core dimensions to extract overlapping or strongly correlated modules of differential molecules across omics. This process will eliminate functionally irrelevant redundant information, ultimately selecting core differential markers that are co-occurring across multiple omics and closely associated with prognostic phenotypes (e.g., survival/death). Data preprocessing will be required prior to model construction: the screened core marker data will be integrated, Z-score standardization will be applied to eliminate dimensional discrepancies across different omics platforms, and the dataset will be randomly partitioned into a training set (for model development) and a test set (for preliminary validation) at a ratio of 8:2 to mitigate overfitting risks at the data level. In this process, samples from eight centers will be selected as training dataset according to the final number of enrolled patients, while the other two centers will act as validation set. The feature dimensionality reduction stage will employ a combined “unsupervised + supervised” strategy: unsupervised dimensionality reduction will first be conducted via principal component analysis (PCA), retaining principal components with a cumulative variance contribution rate > 80% to initially compress feature dimensions; subsequently, least absolute shrinkage and selection oerator (LASSO) will be applied for supervised dimensionality reduction, selecting features with non-zero coefficients to exclude irrelevant variables. For advanced applications, principal component regression (PCR) or partial least squares discriminant analysis (PLS-DA) will be integrated to further prioritize core features strongly correlated with prognostic phenotypes, ultimately refining high-dimensional features to 20–50 variables to balance model complexity and predictive performance. For model construction, appropriate multi-omics fusion strategies will be selected. For early fusion, all core omics features will be integrated first, followed by building a unified predictive model using algorithms such as random forest (RF) or support vector machine (SVM). For late fusion, individual predictive models will be constructed for each omics separately, and the results will then be integrated via weighted voting (e.g., transcriptomics model weight = 0.4, metabolomics = 0.3, proteomics = 0.3) or the Stacking ensemble method to improve prediction stability. Common tools to be utilized include the multi-omics prediction module in MetaboAnalyst and ensemble learning libraries within Python’s scikit-learn. Model performance will be rigorously evaluated using standardized metrics: classification models (e.g., survival/death prognostic prediction) will be quantified using accuracy, sensitivity, specificity, area under the ROC curve (AUC), and confusion matrix. Regression models (e.g., prognostic risk score prediction) will assess fitting efficacy using coefficient of determination (R²), root mean square error (RMSE), and mean absolute error (MAE). Notably, AUC ≥ 0.85 (for classification models) and R² ≥ 0.7 (for regression models) will be established as key criteria for confirming the clinical utility of the model.

### Outcomes

The primary outcome of this study is the 28-day clinical outcome, which includes survival and death. The secondary outcomes include 28-day ICU length of stay and ventilator-free days. For patients with moderate to severe ARDS undergoing invasive mechanical ventilation, additional outcomes include RNA-seq, and scRNA-seq analyses of peripheral blood and BALF will be collected on 24h of inclusion and before extubation. For patients unable to be extubated within 7–14 days, samples will be collected during this period. Respiratory mechanics parameters, severe ultrasound indexes, EIT data, the correlation of physiological parameters will also be evaluated using binary analysis.

### Sample size calculation

The study hypothesizes that the developed model will achieve an area under the ROC curve of at least 0.85 for predicting the 28-day mortality in ARDS patients, and the goal is to test if this value is greater than 0.70, which is considered the threshold for good discriminant ability. The study is designed with α = 0.05 and β = 0.10, and the proportion of lost follow-up or withdrawal is less than 10%. The sample size was calculated using PASS-16 software, which determined that at least 66 deaths must be observed for the study. Referring to previous literature, the 28-day mortality rate of ARDS patients is about 40% [4,7,8], and it is estimated that 165 patients with ARDS need to be enrolled in the study. In addition, given the stringent quality requirements of multi-omics testing for samples and to account for potential test failure rates, we aim to enroll 200 patients to ensure the final testing meets the predetermined sample size requirements.

### Statistical analysis

For continuous variables with a normal distribution, the t-test or analysis of variance will be employed to compare means. For those with a non-normal distribution, the rank sum test will be used to compare medians. A Cox regression model will be constructed to establish a predictive model for 28-day mortality in patients with ARDS. The predictive performance of the model will be evaluated using the area under the receiver operating characteristic (ROC) curve, as well as sensitivity and specificity.

### Ethics and dissemination

This study protocol adheres to the Declaration of Helsinki and the guidelines for the quality management of drug clinical trials. Prior to enrollment, each subject or their legal representative will receive a comprehensive explanation of the study and will be required to sign the informed consent form. For patients without capacity for consent, informed consent shall be obtained from their legal representatives. Once the patient regains capacity for consent, the consent shall be synchronized with the patient themselves. The legal representative shall sign a power of attorney either in advance or simultaneously. Subject data stored in the DMS will be password-protected, with access restricted exclusively to authorized users at the appropriate permission level to ensure the security of data used for statistical analysis. This study has been approved by the Ethics Committee of the PLA General Hospital for the PLA medical consortium (Approval Number: 2022113030901836) as well as the ethics committees of other individual participating centers.

### Quality control

Prior to the start of the study, key researchers have received training on the Drug Clinical trial quality Management Code (GCP). All researchers underwent comprehensive training to ensure study quality, including research protocols, informed consent forms, case reports, standard operating procedures for subject data collection, and methods for biological sample collection and preservation. In addition, a detailed researcher’s manual was developed to ensure compliance with the program. During the study, biological samples will be tested by trained professionals and genetic testing companies.

An executive committee, comprising members of the expert group, including expert committees composed of clinicians, statisticians, and quality managers, will oversee clinical study progress, organize study-related seminars, and manage data collection and analysis. Quality control meetings will be convened as needed.

A dedicated quality control team, composed of qualified inspectors, will conduct research audits and monitoring every two weeks. Inspections will cover the completeness of study documentation and informed consent records, adherence to inclusion/exclusion criteria, accuracy of raw data, management of adverse events (AEs) and serious adverse events (SAEs), sample storage conditions, and data collection records. Findings will be reported to key researchers and relevant committees. Adverse events, such as cardiac events, drug side effects or deaths, will be recorded using data collected for secondary outcomes, such as mortality and hospitalization. Participating facilities must immediately report all AEs to the quality control department. Serious events will be evaluated by the principal researcher or other clinical members of the study management team to determine the cause and prognosis. All incidents will be tracked until resolution or decision to discontinue follow-up, with relevant information shared among researchers.

This study will be conducted across 10 centers, with a comprehensive quality control framework implemented throughout to ensure consistency and compliance. Prior to the formal initiation of data and sample collection, standardized, uniform training will be provided to all personnel involved in the multi-center collaboration. The training content will comprehensively cover key operational protocols, including: rigorous adherence to patient inclusion/exclusion criteria; standardized informed consent procedures; requirements for the accurate collection and complete documentation of clinical data; and protocol-aligned guidelines for sample collection, preprocessing, preservation, and transportation. During study implementation, a dedicated internal communication group will be established for each participating center to facilitate timely reporting of patient enrollment progress and granular sample collection details, thereby ensuring transparency and real-time oversight. Additionally, the study project manager will monthly collect, organize, and review enrollment data and online information submissions, with a primary focus on verifying that all collected samples and completed data comply with preset study requirements. In the event that non-compliant patients or protocol deviations are identified at any center, the principal investigator (PI) of the leading center will provide targeted, timely guidance to address the issue. For centers that repeatedly fail to meet the established standards for sample quality or data documentation, supplementary, needs-specific training will be arranged to rectify gaps and ensure consistent adherence to study protocol. Furthermore, any modifications to the protocol, including changes to study type, design, subject population, sample size or pilot procedure, must undergo formal revision and approved by the Ethics Committee. Updates will be documented in Clinical Trials registry. If a protocol change may affect trial-related risks, the informed consent of the recipient will be re-obtained.

### Patient and public involvement

No patients or members of the public were involved in the design, conduct, reporting, or dissemination of this research study.

## Discussion

ARDS remains a critical challenge in critical care medicine, primarily due to its striking heterogeneity across etiology, pathophysiology, clinical presentation, and imaging characteristics, which has hindered the development of universally effective therapies [4,5,29]. While recent advancements in medical and biological technologies have deepened our understanding of this heterogeneity, significant gaps persist in translating such insights into improved patient outcomes [30]. Against this backdrop, the paradigm of precision medicine has emerged as a transformative approach, emphasizing the need to tailor diagnosis and treatment to individual patient characteristics [31,32]. This shift has underscored the core value of integrating advanced technologies such as molecular biology, bioinformatics, scRNA-seq, RNA-seq, microbiome analysis, proteomics, and metabolomics into ARDS research. These technical approaches hold promise for unraveling dynamic change patterns of key molecules, signaling pathways, and cell subsets during ARDS initiation and progression, ultimately enabling individualized and targeted therapeutic strategies. Such efforts not only represent the future direction of ARDS research but also strive to break through the limitations of the current “one-size-fits-all” treatment model.

The present study protocol contributes to this evolving landscape through several key strengths. It is the first large-scale, multicenter observational cohort study with rigorous quality control, focused on mechanically ventilated patients with moderate to severe ARDS. By systematically collecting comprehensive baseline and clinical data, alongside BALF and peripheral blood samples at two critical time points (day 1 post-admission and pre-extubation), the study protocol enables in-depth multi-omics analyses.

By integrating multi-omics data, the pathophysiological characteristics of ARDS patients with distinct prognoses across multiple dimensions can be systematically studied. For instance, single-cell omics enables high-resolution dissection of cellular subsets, such as alveolar epithelial cells, immune cells, stromal cells, and their dynamic phenotypic shifts during ARDS progression and resolution. This approach allows for the identification of rare cell populations or cell-state transitions that may underlie prognostic differences. Complementarily, transcriptomics facilitates the identification of differentially expressed genes, providing a foundation for deciphering the genetic underpinnings and early molecular regulatory networks driving ARDS pathogenesis. Proteomics focuses on functional alterations of proteins at the post-translational modification level (e.g., phosphorylation and acetylation), while metabolomics captures the dynamic remodeling of metabolite profiles during disease progression. Together, these omics approaches synergistically delineate the rewiring of key pathways linking molecular expression, cellular responses, and metabolic phenotypes in ARDS.

Additionally, microbiomics clarifies the crosstalk between lung microbiota dysbiosis and host immune responses, with insights further refined by single-cell analyses that resolve cell-type-specific immune-microbe interactions. This sheds light on the mechanisms by which microecological imbalances and cellular heterogeneity collectively contribute to prognostic disparities in ARDS. The cross-validation and complementary analysis of multi-omics technologies, anchored by single-cell resolution, not only provide a comprehensive depiction of the intricate regulatory networks among the host (at cellular and molecular levels), microbiota, and environment throughout ARDS progression and resolution, but also enable the identification of key differential molecules, cell-type-specific signaling pathways, and intercellular interaction targets. This multi-dimensional evidence serves to accurately distinguish disease subtypes, predict prognosis, and develop targeted therapeutic strategies, highlighting the pivotal value of integrating single-cell omics with multi-omics approaches in ARDS mechanism research and clinical translation.

Despite its comprehensiveness, the study has inherent limitations. First, as an exploratory study, although 165 patients will be enrolled in the study, the sample size remains relatively limited for high-dimensional multi-omics analyses. Therefore, future studies should consider expanding the sample size in independent cohorts for further validation. Second, while the multicenter design enhances the external validity of the results, it also introduces challenges related to ensuring consistency across sites, particularly in data collection, sample processing, and outcome assessment across sites, which must be rigorously addressed to maintain result reliability. Furthermore, multi-omics profiling entails substantial costs, which requires substantial research funding support. Looking forward, longitudinal studies are warranted to evaluate long-term outcomes of ARDS survivors and assess the durability of any predictive models derived from the current data. Additionally, interventional studies are critical to translating the study’s findings into clinical practice, testing whether targeting identified molecular or microbial signatures improves patient outcomes. Such follow-up research will enhance the external validity of our results and advance the collective goal of refining ARDS management and prognosis.

Another potential limitation of the present study is the exclusive focus on patients with moderate-to-severe ARDS. This design was deliberately adopted to align with the research objective of targeting high mortality in severe disease, as moderate-to-severe ARDS is associated with a significantly higher clinical burden and poorer outcomes. However, this focus inherently restricts the generalizability of our findings to the full ARDS spectrum, particularly excluding patients with mild ARDS. Consequently, our results may not capture the factors driving disease progression from mild to severe stages, nor can they inform clinical decision-making for the mild ARDS population. Future studies encompassing a broader range of ARDS severity grades are warranted to clarify the predictive factors of disease progression and validate the applicability of our findings across the entire spectrum of ARDS. Furthermore, for specific omics data, particularly transcriptomic findings, targeted validation approaches such as probe-based assays or reverse transcription-polymerase chain reaction (RT-PCR) can be used to confirm the reliability and biological relevance of key candidate genes or pathways.

In summary, this study leverages a rigorous multicenter design and multi-omics approaches to address critical knowledge gaps in ARDS onset and progression. By focusing on host-microbe interactions, and predictive biomarkers, it aligns with the precision medicine agenda and paves the way for more effective, personalized care in this challenging patient population.

### Study status

The first patient was recruited on November 15, 2023, and is expected to be completed by November 2025. Current protocol version is 1.1.

## Supporting information

S1 File Informed consent. Informed consent document for patients to sign upon enrollment.
(DOCX)

S2 File Case report form for day1. Case report form for the first day of enrollment.
(DOCX)

S3 File Case report form for day3. Case report form for the third day of enrollment.
(DOCX)

S4 File Case report form for day2 to day28. Case report form for days 1–28, excluding day 1 and day 3.
(DOCX)

S5 File Methods for multi-omics. Methodological description of the multi-omics detection approaches to be used.
(DOCX)
