# Supplementary material for: Multi-modal data to identify key factors influencing lung injury in ARDS patients undergoing invasive mechanical ventilation: A prospective multi-center observational study protocol
Source: PLoS One. 2026 Jan 23;21(1):e0332985. doi: 10.1371/journal.pone.0332985 (PMC12829816; doi:10.1371/journal.pone.0332985)
Supplement: S1 File — Informed consent document for patients to sign upon enrollment. (DOCX) [file pone.0332985.s001.docx]

**Additional file 1: Informed consent**

**Informed consent**

(Version Number: V1.0; Version Date: September 30, 2022)

**Dear volunteers or legal representatives:**

We cordially invite you and your family member to participate in a study titled “A Cohort Study on the Factors Influencing Lung Injury in ARDS Patients Undergoing Invasive Mechanical Ventilation”, which is part of research on key repair mechanisms and treatment systems following viral-induced acute lung injury. This research will be conducted at the Department of Respiratory and Critical Medicine of the PLA General Hospital, in collaboration with participating units. The study has received review and approval from the Medical Ethics Committee of the PLA General Hospital.

Prior to making a decision on whether you or your family member wants to participate in this study, it is crucial to comprehend the rationale and implications of participate. We urge you to meticulously review the provided information and consult with friends, family members, and personal physicians as needed. A research physician or members of the research team will also provide an explanation of the study. Please ensure that all your questions are addressed by the research physician or staff before making a decision and signing this informed consent form, and do not hesitate to request additional details if required.

Even if you wish for your family member to participate in this study, your family member may not meet the eligibility criteria. If you or your family member is not eligible to participate, the research physician or study staff will discuss the reasons with you.

**Research Background**

Acute Respiratory Distress Syndrome (ARDS) is a clinical syndrome characterized by acute hypoxic respiratory failure resulting from pulmonary inflammation rather than cardiogenic pulmonary edema. ARDS is an acute, diffuse, inflammatory lung injury triggered by predisposing risk factors such as pneumonia, non-pulmonary infection, trauma, transfusion, burn, aspiration or shock. This injury leads to increased pulmonary vascular and epithelial permeability, lung edema and gravity-dependent atelectasis, collectively contributing to the loss of aerated lung tissue.

The clinical hallmarks of ARDS include arterial hypoxemia and diffuse radiographic opacities, accompanied by increased intrapulmonary shunt, increased alveolar dead space and decreased lung compliance. Its clinical presentation is further influenced by medical interventions, such as positioning, sedation, paralysis, positive end-expiratory airway pressure and fluid balance. Histological findings vary and may include intra-alveolar edema, inflammation, hyaline membrane formation and alveolar hemorrhage.

ARDS, as a critical clinical manifestation of severe acute lung injury, has become a critical topic in clinical research due to its rapid progression, high mortality rate, and poor prognosis. Electrical impedance tomography (EIT) has been used to evaluate the degree of pulmonary collapse in gravity-dependent regions in patients with COVID-19 secondary to ARDS. Studies have shown that the degree of collapse in gravity-dependent regions in the supine position was significantly related to the improvement of oxygenation in the prone position, which could accurately predict the therapeutic effect of prone position ventilation. The application of transpulmonary pressure is recognized as one of the methods of titrating individualized positive end-expiratory pressure (PEEP) in patients with ARDS, which can avoid lung injury caused by stress and strain, improve clinical symptoms, predict clinical outcome and improve the survival rate of patients. However, the titration of PEEP using transpulmonary pressure is based on a single-compartment model, which has inherent limitations. Given the significant heterogeneity of ARDS patients, there is ongoing debate on how to achieve truly individualized PEEP titration.

At present, the clinical diagnosis and evaluation of ARDS mainly depend on the clinical features and chest imaging findings. Critical ultrasound can monitor the changes in lung ventilation in ARDS patients in real-time. Utilizing the lung ultrasound scores (LUS), it enables dynamic monitoring of changes of pulmonary ventilation during the lung recruitment process, facilitating the personalized adjustment of mechanical ventilation parameters. Furthermore, it can assess the efficacy of prone positioning and predict the clinical outcomes for ARDS patients. The hallmark histopathological feature of ARDS is diffuse alveolar damage. Inflammatory cytokine storms and immune dysregulation are the primary pathophysiological manifestations of ARDS. Despite extensive research, significant advancements in understanding the role of biomarker expression in the pathogenesis, diagnosis, and pathophysiology-based therapeutic selection of ARDS have yet to be achieved. The quantitative evaluation of the entire ARDS process remains in its experimental phase. Consequently, identifying distinct clinical phenotypes based on disease-specific endotypes represents a promising direction for future development.

**Objectives**

(1) It is hypothesized that the interaction between the respiratory microbiota and host immunity modulates immune function, thereby influencing patient outcomes. By employing metagenomic sequencing and other advanced methodologies, we aim to elucidate the mechanisms underlying the interaction between respiratory microbiota and host immunity.

(2) To further analyze immune tolerance and immune exhaustion by employing transcriptome sequencing and single-cell sequencing in patients with diverse outcomes, including both survivors and non-survivors, as well as those experiencing primary versus recurrent infections.

(3) To compare differential metabolites in plasma and bronchoalveolar lavage fluid (BALF) from patients with different survival outcomes using metabolomics. Several prognostically significant biomarkers will be identified for subsequent experimental validation and mechanistic studies using a mouse model of ARDS.

(4) To establish the relationship between clinical indicators and phages in the lower respiratory tract, by comparing differences in parameters such as white blood cells, neutrophils, C-reactive protein (CRP), interleukin-6 (IL-6), procalcitonin (PCT), lactate dehydrogenase (LDH), lactic acid, and cytokines in BALF from patients with different outcomes.

To elucidate the structural characteristics of phage communities in the lower respiratory tract of patients with moderate to severe ARDS, and to compare differences in phage types, abundance, and antibiotic resistance genes between patients with different outcomes, through utilizing Next-Generation Metagenomic Sequencing.

To explore the differences in phage-bacterial interaction networks and mechanisms in the lower respiratory tract of patients with different outcomes, and to attempt to establish a correlation between the lower respiratory tract phageome and disease prognosis, by employing CRISPR and Machine Learning techniques.

(5) To implement hemodynamics-guided PEEP titration by utilizing pulmonary artery floating catheters (Swan-Ganz Catheter).

(6) To monitor the changes in respiratory therapy-related parameters during the course of moderate to severe ARDS patients.

(7) To observe the characteristics of lung injury changes with the progression of the disease, and dynamically evaluate the relationship between lung injury and clinical outcomes.

(8) To provide clinical data support for the development of prediction models for acute lung injury and other organ injuries.

**Range**

Patients with moderate and severe ARDS undergoing invasive mechanical ventilation hospitalized in the Respiratory Intensive Care Unit (RICU), Medical Intensive Care Unit (MICU) or Emergency Intensive Care Unit (EICU) of Chinese PLA General Hospital and participating units.

**Research overview**

(1) Patient Enrollment: The plan is to recruit 200 patients with moderate and severe ARDS undergoing invasive mechanical ventilation from the RICU, MICU and EICU admissions at the General Hospital of the PLA and participating units.

(2) Baseline Data Collection: This includes basic patient information (e.g. hospital number, name, gender, age, nationality, etc.), medical history (e.g. smoking history, body mass index, APACHE II score, SOFA score, lung disease history, heart disease history, hypertension history, diabetes history, etc.), and risk factors related to ARDS.

(3) Sample Collection: Samples will be collected, including at least 6 mL of peripheral blood each in non-anticoagulant (red-yellow) tubes and anticoagulant (purple) tubes, 10-15 mL of BALF for proteomic, metabolomic, immunological, genomic, and other analyses, on the first 24 hours after enrollment and before extubation (for patients who fail to extubate within 7 to14 days, specimens will be collected during this period).

(4) Dynamic Monitoring: Monitor the fluctuations in relevant indicators of patients, as well as primary outcome (28-day survival or mortality) and secondary outcome measures, to determine whether there are statistical significance differences.

(5) Collection of Clinical Data During Acute and Recovery Phases

a. Laboratory and microbiological tests are conducted on specific days following inclusion (d1, d3±1, d7±1, d14±2, d21±2, d28±2), and prior to extubation, discharge, or death. The tests encompass a range of inflammatory markers such as white blood cell count (WBC), neutrophil count and proportion, interleukin-6 (IL-6), C-reactive protein (CRP), procalcitonin (PCT), along with assessments of coagulation, blood sugar, blood lipids, electrolytes, cardiac enzymes, immune indicators, BALF cell count and differential examination.

b. Respiratory mechanics assessed on day1 after inclusion and before extubation primarily consist of the respiratory system compliance (C_rs_), static compliance of the lungs (C_lung_), respiratory system airway resistance (R_rs_), esophageal pressure (P_es_), transpulmonary pressure (P_tp_), transpulmonary driving pressure (△P_tp_), driving pressure (△P), dead space fraction (V_D_/V_t_) and mechanical power (MP).

c. Arterial blood gases measured on the same schedule as the laboratory tests (d1, d3±1, d7±1, d14±2, d21±2, d28±2, before extubation, discharge, or death).

d. Ventilation perfusion distribution assessed on day1 following inclusion and before extubation, this includes Ventilation partition (ROL), central of ventilation (COV), global inhomogeneity index (GI), EIT perfusion value.

e. Cardiac ultrasound performed on day1 following inclusion and before extubation. Using a phased array probe to measure left ventricular ejection fraction (long-axis view of the chest) and the diameter of the inferior vena cava during inspiration and expiration (four-chamber view of the subxiphoid).

f. Lung ultrasound score conducted on day1 following inclusion and before extubation.

g. Mechanical ventilation parameters recorded on day1 following inclusion and just prior to extubation, these parameters include ventilation mode, inhaled oxygen fraction (FiO_2_), positive end-expiratory pressure (PEEP), respiratory rate (f), tidal volume (V_T_), flow rate, expiratory tidal volume (V_te_), peak pressure (P_peak_), plateau pressure (P_plat_) and airway resistance (R_AW_).

h. PICCO parameters assessed on day 1 following inclusion and before extubation, including central venous pressure (CVP), cardiac index (CI), global end-diastolic index (GEDI), extravascular lung water index (ELWI), pulmonary vascular permeability index (PVPI), systemic vascular resistance index (SVRI), and global ejection fraction (GEF).

j. Clinical indicators recorded on day 1 following inclusion and before extubation, these include heart rate, blood pressure, oxygen saturation (SpO_2_), Acute physiology and chronic health evaluation (APACHE)II score, and Sequential Organ Failure Assessment (SOFA)score.

k. Document the medication use within 28 days, including antibiotics, neuromuscular blocking agents, vasoactive drugs, hormones, and immunosuppressants.

(6) Observational Outcomes

a. Primary Outcome: The primary outcome is the clinical outcome within 28 days.

b. Secondary Outcomes: Assess the correlation between patient demographic data, clinical data, and proteomic indicators, respiratory mechanics parameters, critical ultrasound indicators, EIT data, and physiological parameters during the acute phase of mechanical ventilation in ARDS and the recovery phase before extubation, using binary analysis.

**The research program**

In this study, the vital signs and laboratory tests of you or your family member during hospitalization will be counted, and your family members will be comprehensively evaluated by bronchoscopy intermittently after inclusion, and each examination time will not exceed half an hour.

**Other treatment options**

You can choose

- Do not participate in this study and continue your routine treatment.
- To participate in other studies.

Please consult with your doctor about your decision.

**Possible impacts of this study**

There is no additional examination in this study and there will be no other adverse effects on you or your family member. If you have any questions about the tests and procedures in the study, you can consult your research physician.

**Risks and adverse reactions of the study**

This study is mainly conducted through bronchoscopy and venous blood sampling. Bronchoscopy is a safe procedure with a low risk of allergic reaction and is harmless to the human body. During the procedure, your family member may experience some mild discomfort.

You will need to tell your family or close friends that your family member is participating in a clinical study. If they have questions about your participation in the study, you can tell them how to contact your family's study physician.

**Research benefits**

Participation in this study may improve you or your family member’s health.

The information obtained from this study will be helpful to assist your or your family member’s diagnosis and treatment process and play an early warning role in related complications.

Relevant research information and results obtained in this study will be informed to you in due course.

**Compensation or reward**

You will not receive any remuneration for participating in this study. However, to compensate for any inconvenience that may be caused by your participation, a free Metagenomic Next Generation Sequencing (m-NGS) test of BALF will be conducted on the first day after inclusion.

**Medical expenses compensation for injuries caused by the research**

If your or your family member’s health does suffer research-related damage as a result of participating in this study, please inform the research physician immediately, who will be responsible for taking appropriate treatment measures for you or your family member.

Even if you have signed this informed consent form, you still retain all your legal rights. If your rights and interests are violated, you can contact the Medical Ethics Committee of the PLA General Hospital, Tel: 010-66937166.

**Confidentiality**

Your or your family member’s medical records will be kept in the hospital, and researchers, research authorities, ethics committees will be allowed to access the medical records. The personal identity will not be disclosed in any public report of the results of this study. We will make every effort to protect the privacy of the personal medical data within the scope permitted by law.

Personal and medical information about you or your family member will be kept confidential and kept in a safe and secure place. At any time, you can request access to your personal information (such as your name and address) and modify it if necessary.

By signing this informed consent form, you agree to allow your or your family member’s personal and medical information to be used for the purposes described above.

**Voluntary**

Participation in this study is completely voluntary. You can refuse to participate in the study or choose to withdraw from the study at any time in the course of the study without any reason. This decision will not affect your or your family member’s future treatment.

If you decide to withdraw from this study, please inform your or your family member’s research doctor in advance. In order to ensure your or your family member’s safety, your or your family member may be required to conduct relevant examinations, which is beneficial to the protection of your or your family member’s health. Please keep this informed consent form.

**Subject consent statement**

By signing below, you confirm that you have read and understood this informed consent form.

I declare:

I have enough time to ask questions related to this study, and my questions have been satisfactorily answered.

I understand that I am/my family member is volunteering to participate in this study. I can withdraw from the study at any time without punishment and without any loss of any benefits or medical services to which I am entitled.

My personal/My family member’s health information can be used and transmitted as described above, and can be added to the research database.

For the following purposes, researchers or other companies and individuals working for or working with researchers my use my or my family member’s personal information, medical records and pathological specimens for future studies (with approval from the Ethics Committee) beyond this study:

- Developing diagnostic technologies to better understanding of safety and efficacy.
- Investigating alternative therapies for patients.
- Gaining a deeper understanding of the diseases involved in the study.
- Improving the efficiency, design, and methodology of future clinical researches.

I agree that my/my family member’s personal physician will be informed that I am/ my family member is participating in this study and that they can provide information about my health to the research physician.

I understand that I will not lose any legitimate rights and interests as a result of signing this informed consent form.

I will receive a signed and dated copy of the informed consent form.

By signing, I agree to participate in the research.

Subject signature:

Signature of subjects: Date:

Name (in block letters): Subject Contact number:

Signature of legal representative (if applicable): Date:

Legal representative’ name (in block letters):

**Statement by the investigators**

I confirm that the details of the study, particularly the possible risks and benefits of participating in the study, were explained to the patient.

Investigator's signature: Date:

Investigator's name (in block letters): Investigator's Contact number:
