# Supplementary material for: Multi-modal data to identify key factors influencing lung injury in ARDS patients undergoing invasive mechanical ventilation: A prospective multi-center observational study protocol
Source: PLoS One. 2026 Jan 23;21(1):e0332985. doi: 10.1371/journal.pone.0332985 (PMC12829816; doi:10.1371/journal.pone.0332985)
Supplement: S3 File — Case report form for the third day of enrollment. (DOCX) [file pone.0332985.s003.docx]

**CRF (Version Number: V1.0 Version Date: September 30, 2022)**

**Cohort Study on Factors of Lung Injury in ARDS Patients Undergoing Invasive Mechanical Ventilation**

**Case Report Form D3±1, D7±1, Pre-extubation, D14±2, D21±2, D28±2, Death/Discharge**

Subject ID: □□-□□□-□□□□

Name Abbreviation: □□□□

Visit Date: □□□□Year □□ Month□□Day

Following Physician:

**Main Symptoms and Examination Information**


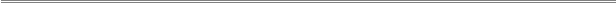


**Description of patient's main symptoms:**

🞎Cough 🞎Copious Sputum 🞎Wheezing/Dyspnea 🞎Hemoptysis 🞎 Fever

🞎 Other

**X-ray:**  ○ Yes Date: ○ No

**CT-Scan:**  ○ Yes Date: ○ No

**Left Lung:** 🞎 Upper Lobe (Superior vision) 🞎 Lingula 🞎 Lower Lobe

Characteristics: 🞎 Consolidation 🞎 Patchy Opacities 🞎 Ground-Glass Opacity

🞎 Reticulation 🞎 Honeycombing 🞎 Linear pacities 🞎Nodules 🞎 patcky

🞎 Emphysema  🞎 Bullae 🞎 Bronchiectasis

🞎 Pleural Effusion ○Yes ○Small  ○ Moderate  ○ Large

**Right Lung**:  🞎 Upper Lobe (Superior vision) 🞎 Lingula 🞎 Lower Lobe

Characteristics: 🞎 Consolidation 🞎 Patchy Opacities 🞎 Ground-Glass Opacity

🞎 Reticulation 🞎 Honeycombing 🞎 Linear pacities 🞎Nodules 🞎 patcky

🞎 Emphysema  🞎 Bullae 🞎 Bronchiectasis

🞎 Pleural Effusion ○Yes ○Small  ○ Moderate  ○ Large

**Vital Signs, Oxygen Therapy, Mechanical Ventilation, etc. Record Sheet**


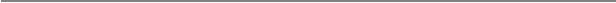


| **Parameters** | | | **Value** | **Unit** |
| --- | --- | --- | --- | --- |
| **Vital Signs** | | Blood Pressure |  | mmHg |
|  |  | Mean Arterial Pressure (1/2 SBP + 2/3 DBP) |  | mmHg |
|  |  | Pulse |  | /min |
|  |  | Heart Rate |  | /min |
|  |  | Respiratory Rate |  | /min |
|  |  | SpO2 |  | % |
|  |  | CVP | ○ No  ○ Yes | cmH₂O |
|  |  | Temperature |  | ℃ |
| **Ventilator Settings** | | | | |
| **Pressure Control Ventilation (PCV)** | Respiratory Rate (F) | |  | /min |
|  | Control Pressure (PC) | |  | cmH₂O |
|  | Inspiratory Time (Ti) | |  | s |
|  | Positive End-Expiratory Pressure (PEEP) | |  | cmH₂O |
|  | Fraction of inspired Oxygen (FiO₂) | |  | % |
| **Volume Control Ventilation (VCV)** | Respiratory Rate (F) | |  | /min |
|  | Tidal Volume (Vt) | |  | ml |
|  | Flow Rate (V) | |  |  |
|  | Inspiratory Hold Time (Tplat) / Inspiratory Time | |  | s |
|  | Positive End-Expiratory Pressure (PEEP) | |  | cmH₂O |
|  | Fraction of inspired Oxygen (FiO₂) | |  | % |
| **Volume-Synchronized Intermittent Mandatory Ventilation (VC-SIMV)** | Respiratory Rate (F) | |  | /min |
|  | Tidal Volume (VT) | |  | ml |
|  | Flow Rate (V) | |  |  |
|  | Inspiratory Hold Time (Tplat) / Inspiratory Time | |  | s |
|  | Pressure Support (PS) | |  | cmH₂O |
|  | Positive End-Expiratory Pressure (PEEP) | |  | cmH₂O |
|  | Fraction of inspired Oxygen (FiO₂) | |  | % |
| **Pressure-Synchronized Intermittent Mandatory Ventilation (PC-SIMV)** | Respiratory Rate (F) | |  | /min |
|  | Control Pressure (PC) | |  | cmH₂O |
|  | Inspiratory Time (Ti) | |  | s |
|  | Pressure Support (PS) | |  | cmH₂O |
|  | Positive End-Expiratory Pressure (PEEP) | |  | cmH₂O |
|  | Fraction of inspired Oxygen (FiO₂) | |  | % |
| **Airway Pressure Release Ventilation (APRV)** | High Pressure (Pₘₐₓ) | |  | cmH₂O |
|  | Low Pressure (Pₘᵢₙ) | |  | cmH₂O |
|  | High Pressure Phase Time (Tₘₐₓ) | |  | s |
|  | Low Pressure Phase Time (Tₘᵢₙ) | |  | s |
|  | Pressure Support (PS) | |  | cmH₂O |
|  | Fraction of inspired Oxygen (FiO₂) | |  | % |
| **Pressure Regulated Volume Control Ventilation (PRVC)** | Fraction of inspired Oxygen (FiO₂) | |  | % |
|  | Positive End-Expiratory Pressure (PEEP) | |  | cmH₂O |
|  | Target Tidal Volume (Vt) | |  | ml |
|  | Respiratory Rate (F) | |  | /min |
|  | Inspiratory Time (Ti) / Inspiratory:Expiratory Ratio | |  | s |
| **Pressure Support Ventilation (CPAP/PSV)** | Pressure Support (PS) | |  | cmH₂O |
|  | Positive End-Expiratory Pressure (PEEP) | |  | cmH₂O |
|  | Fraction of inspired Oxygen (FiO₂) | |  | % |
| PEEP Titration Method: ○ ARDSnet method ○ EIT method ○ Transpulmonary pressure method  ○ Other | | | | |
| PEEP Value |  | |  | cmH₂O |
| Prone Positioning | ○ No ○ Yes Time (h/d) | | | |
| **End-tidal CO₂ Monitoring Parameters** | Actual Tidal Volume (V_te_) | |  | ml |
|  | Actual Respiratory Rate (RR) | |  | /min |
|  | End-tidal CO₂ (etCO₂) | |  |  |
|  | Dead Space Ratio (VD/VT) | |  |  |
| **Respiratory Mechanics Parameters**  **(**Measured in dedicated mode: VCV, f 12/min, VT 6ml/Kg, flow 30L/min, I:E 1:2) | Peak Airway Pressure (P_peak_) | |  | cmH₂O |
|  | Plateau Pressure (P_plat_) | |  | cmH₂O |
|  | Airway Resistance (Raw) | |  |  |
|  | Respiratory System Compliance (C_rs_) | |  |  |
| **EIT** | Global Inhomogeneity Index (GI) | |  |  |
|  | Shunt Fraction (Qs/Qt) | |  |  |
|  | EIT Perfusion Value | |  |  |
| **Transpulmonary Pressure** | End-inspiratory Esophageal Pressure (P_es_plat) | |  | cmH₂O |
|  | End-expiratory Esophageal Pressure (P_es_PEEP) | |  | cmH₂O |
|  | End-inspiratory Transpulmonary Pressure (P_tp_plat) | |  | cmH₂O |
|  | End-expiratory Transpulmonary Pressure (P_tp_PEEP) | |  | cmH₂O |
|  | Lung Compliance (Clung) | |  |  |
|  | Chest Wall Compliance (CCW) | |  |  |
| **Echocardiography** | Cardiac Output | |  |  |
| **Lung Ultrasound** | Location & Score | | Left Lung | Right  Lung |
| **0 points: A-lines or B-lines < 2;**  **1 point: Few B-lines (spacing >7 mm)*);**  **2 points: Numerous B-lines (spacing <3mm);**  **3 points: Consolidation (pleural effusion, bronchogram)** | Upper BLUE point | |  |  |
|  | Lower BLUE point | |  |  |
|  | Diaphragmatic point | |  |  |
|  | PLAPS point | |  |  |
|  | Posterior BLUE point | |  |  |
|  | Total Score | |  |  |
| **Diaphragm** | Respiratory Support Method | |  |  |
|  | Presence of Spontaneous Breathing | |  |  |
|  | Diaphragm Excursion | |  |  |
|  | Ti | |  | s |
|  | Contraction Velocity | |  |  |
|  | E-T Index | |  |  |
|  | Diaphragm Thickness | |  |  |
|  | Diaphragm Thickening Fraction | |  |  |
| **Inferior Vena Cava (IVC)** | Diameter (Inspiration-Expiration) | |  |  |
|  | IVC Collapsibility Index | |  |  |
| **PiCCO** | CVP (8-12) | |  | cmH₂O |
|  | Cardiac Index (CI) (3-5) | |  | L/min/m² |
|  | Global End-Diastolic Volume Index (GEDI) (680-800) | |  | ml/m² |
|  | Extravascular Lung Water Index (ELWI) (3-7) | |  | ml/kg |
|  | Pulmonary Vascular Permeability Index (PVPI) (1-3) | |  |  |
|  | Systemic Vascular Resistance Index (SVRI) (1700-2400) | |  | Dyn·s·cm⁻⁵·m² |
|  | Global Ejection Fraction (GEF) (25%-35%) | |  | % |

Note: Red-labeled items are required at D3/D7/pre-extubation

**Blood Biochemistry and Infection Markers (Date: )**

| **Testing** | **Indexes** | **Value** | **Normal Range** | **Unit** | **Clinical Significance** |
| --- | --- | --- | --- | --- | --- |
| **Liver Function** | Aspartate Aminotransferase (AST) |  | 0-40 | U/L | □1 □2 □3 □4 |
|  | Alanine Aminotransferase (ALT) |  | 0-40 | U/L | □1 □2 □3 □4 |
|  | Total Bilirubin (TBIL) |  | 0-21 | μmol/L | □1 □2 □3 □4 |
|  | Direct Bilirubin (DBIL) |  | 0-8.6 | μmol/L | □1 □2 □3 □4 |
|  | Albumin (ALB) |  | 35-50 | g/L | □1 □2 □3 □4 |
|  | Total Protein (TP) |  | 55-80 | g/L | □1 □2 □3 □4 |
| **Kidney Function** | Blood Urea Nitrogen (BUN) |  | 1.8-7.5 | mmol/L | □1 □2 □3 □4 |
|  | Creatinine (Cr) |  | 57-111 | μmol/L | □1 □2 □3 □4 |
|  | Uric Acid (UA) |  | 208-428 | μmol/L | □1 □2 □3 □4 |
| **Electrolytes** | Calcium (Ca) |  | 2.09-2.54 | mmol/L | □1 □2 □3 □4 |
|  | Magnesium (Mg) |  | 0.6-1.4 | mmol/L | □1 □2 □3 □4 |
|  | Sodium (Na) |  | 130-150 | mmol/L | □1 □2 □3 □4 |
|  | Potassium (K) |  | 3.5-5.5 | mmol/L | □1 □2 □3 □4 |
|  | Chloride (Cl) |  | 94-110 | mmol/L | □1 □2 □3 □4 |
|  | Phosphorus (P) |  | 0.86-1.6 | mmol/L | □1 □2 □3 □4 |
| **Cardiac Markers** | Lactate Dehydrogenase (LDH) |  | 40-250 | U/L | □1 □2 □3 □4 |
|  | Troponin (cTnI/cTnT) |  | 0-0.1 | ng/ml | □1 □2 □3 □4 |
|  | Creatine Kinase (CK) |  | 2-200 | U/L | □1 □2 □3 □4 |
|  | Creatine Kinase MB Isoenzyme (CK-MB) |  | 0-6.5 | ng/ml | □1 □2 □3 □4 |
|  | B-type Natriuretic Peptide (BNP) |  | 0-150 | pg/ml | □1 □2 □3 □4 |
| **Lipids** | Total Cholesterol (TC) |  | 3.1-5.7 | mmol/L | □1 □2 □3 □4 |
| **Glucose etc.** | Fasting Blood Glucose (FBG) |  | 3.4-6.1 | mmol/L | □1 □2 □3 □4 |
|  | Beta-Hydroxybutyrate (D-3-Hydroxybutyrate) |  |  |  | □1 □2 □3 □4 |
|  | Fasting Insulin |  |  |  | □1 □2 □3 □4 |
| **Blood Routine** | White Blood Cell Count (WBC) (10⁹/L) |  | 3.5-10 |  | □1 □2 □3 □4 |
|  | Neutrophil Count |  |  |  | □1 □2 □3 □4 |
|  | Neutrophil Percentage (%) |  | 50-70 |  | □1 □2 □3 □4 |
|  | Lymphocyte Percentage (%) |  | 20-40 |  | □1 □2 □3 □4 |
|  | Monocyte Percentage (%) |  |  |  | □1 □2 □3 □4 |
|  | Platelet Count (PLT) (10⁹/L) |  | 100-300 |  | □1 □2 □3 □4 |
| **Infection Markers** | Interleukin-6 (IL-6) (pg/ml) |  | 0-5.9 | pg/ml | □1 □2 □3 □4 |
|  | C-Reactive Protein (CRP) (mg/dl) |  | 0-0.8 | mg/dl | □1 □2 □3 □4 |
|  | Procalcitonin (PCT) (ng/ml) |  | <0.5 | ng/ml | □1 □2 □3 □4 |
|  | Ferritin |  | 30-400 | ng/ml | □1 □2 □3 □4 |
|  | Interferon-gamma (IFN-γ) |  |  |  | □1 □2 □3 □4 |
|  | Interleukin-10 (IL-10) |  | 0-9.1 | pg/ml | □1 □2 □3 □4 |
|  | Interleukin-1β (IL-1β) |  | 0-5 | pg/ml | □1 □2 □3 □4 |
|  | Tumor Necrosis Factor-alpha (TNF-a) |  | 0-8.1 | pg/ml | □1 □2 □3 □4 |
| **Cellular Immunity** | Total T Cell Count |  | 955-2860 | cells/μl | □1 □2 □3 □4 |
|  | CD4+ T Cells |  | 550-1440 | cells/μl | □1 □2 □3 □4 |
|  | CD8+ T Cells |  | 320-1250 | cells/μl | □1 □2 □3 □4 |
|  | B Cell Count |  | 90-560 | cells/μl | □1 □2 □3 □4 |
|  | NK Cell Count |  | 150-1100 | cells/μl | □1 □2 □3 □4 |
|  | CD4+/CD8+ Ratio |  | 0.71-2.78 |  | □1 □2 □3 □4 |
| **Humoral Immunity** | Immunoglobulin G (IgG) |  | 700-1600 | mg/dl | □1 □2 □3 □4 |
|  | Immunoglobulin M (IgM) |  | 40-230 | mg/dl | □1 □2 □3 □4 |
|  | Immunoglobulin A (IgA) |  | 70-400 | mg/dl | □1 □2 □3 □4 |
|  | Immunoglobulin E (IgE) |  | 0-100 | IU/ml | □1 □2 □3 □4 |
| **Coagulation Function** | Prothrombin Time (PT) |  | 15-21 | s | □1 □2 □3 □4 |
|  | Activated Partial Thromboplastin Time (APTT) |  | 30-45 | s | □1 □2 □3 □4 |
|  | Fibrinogen (Fib) |  | 2-4 | g/L | □1 □2 □3 □4 |
|  | D-dimer |  | 0-0.5 | μg/ml | □1 □2 □3 □4 |
| **Blood Gas Analysis** | pH |  | 7.35-7.45 |  | □1 □2 □3 □4 |
|  | PaO₂ |  | 80-100 | mmHg | □1 □2 □3 □4 |
|  | PaCO₂ |  | 35-45 | mmHg | □1 □2 □3 □4 |
|  | PaO₂/FiO₂ |  | 400-500 |  | □1 □2 □3 □4 |
|  | Lactate (Lac) |  | 0.7-2.1 | mmol/L | □1 □2 □3 □4 |
|  | Actual Bicarbonate (HCO₃⁻) |  | 20-26 | mmol/L | □1 □2 □3 □4 |
| **Fungal** | BALF Galactomannan (GM) |  | >0.65 |  | □1 □2 □3 □4 |
|  | (1,3)-β-D-Glucan (G Test) |  | 0-100 | pg/ml | □1 □2 □3 □4 |
|  | Galactomannan (GM Test) |  | <0.5 Neg; ≥0.5 Pos |  | □1 □2 □3 □4 |
| **Bronchoalveolar Lavage Fluid (BALF)** | Macrophage Percentage |  | >84 | % | □1 □2 □3 □4 |
|  | Neutrophil Percentage |  | <3 | % | □1 □2 □3 □4 |
|  | Lymphocyte Percentage |  | <13 | % | □1 □2 □3 □4 |
|  | Eosinophil Percentage |  | 0 | % | □1 □2 □3 □4 |
|  | Total Cell Count |  | <13×10⁶/L | cells/L | □1 □2 □3 □4 |

(Note: 1=Normal; 2=Abnormal but no clinical significance; 3=Abnormal with clinical significance; 4=Not tested)

**APACHE II Score**

1. **Acute Physiology Score = ( ) points**

| **Parameters** | **Score-Value** | | | | | **Value** | **Score** |
| --- | --- | --- | --- | --- | --- | --- | --- |
|  | 0 | 1 | 2 | 3 | 4 |  |  |
| 1. Temperature (T) (°C) | 36.0-38.4 | 34.0-35.9 38.5-38.9 | 32.0-33.9 | 30.0-31.9 39.0-40.9 | ≤29.9 ≥41.0 |  |  |
| 2. Mean Arterial Pressure (MAP) (mmHg) | 70-109 |  | 50-69 110-129 | 130-159 | ≤49 ≥160 |  |  |
| 3. Heart Rate (HR) (/min) | 70-109 |  | 55-69 110-139 | 40-54 140-179 | ≤39 ≥180 |  |  |
| 4. Respiratory Rate (RR) (/min) | 12-24 | 10-11 25-34 | 6-9 | 35-49 | ≤5 ≥50 |  |  |
| 5. Pulmonary Oxygenation Function (mmHg):  If FiO₂ >50%, use A-aDO₂; If FiO₂ <50%, use PaO₂ | >70 <200 | 61-70 | 200-349 | 55-60 350-499 | <55 ≥500 |  |  |
| 6. Arterial blood pH or HCO₃⁻ (mmol/L) | 7.33-7.49  22-31.9 | 7.50-7.59  32-40.9 | 7.25-7.32  18-21.9 | 7.15-7.24 7.60-7.69  41-51.9 15-17.9 | <7.15 ≥7.70  <15 ≥52 |  |  |
| 7. Na⁺ (mmol/L) | 130-149 | 150-154 | 120-129 155-159 | 111-119 160-179 | ≤110 ≥180 |  |  |
| 8. K⁺ (mmol/L) | 3.5-5.4 | 3.0-3.4 5.5-5.9 | 2.5-2.9 | 6.0-6.9 | <2.5 ≥7.0 |  |  |
| 9. Serum Creatinine Cr (μmol/L)  Multiply score by 2 for AKI | 53.04-123.76 |  | <53.04 132.6-167.96 | 176.80-300.56 | ≥309.40 |  |  |
| 10. Hematocrit (Hct) (%) | 30.0-45.9 | 46.0-49.9 | 20.0-29.9 50.0-59.9 |  | <20 ≥60 |  |  |
| 11. White Blood Cell Count (WBC) (10⁹/L) | 3.0-14.9 | 15.0-19.9 | 1.0-2.9 20.0-29.9 |  | <1.0 ≥40 |  |  |
| 12. GCS Score | E: | V: | M: | Actual GCS= | 15 - Actual GCS = ( ) points | | |

**Notes**:

1. MAP (mmHg) = 1/3 SBP + 2/3 DBP
2. Pulmonary Oxygenation Function:
   - If FiO₂ <50%, use PaO₂ (mmHg).
   - If FiO₂ >50%, use Alveolar-arterial oxygen difference [A-aDO₂].
     A-aDO₂ = FiO₂ × (PB - PH₂O) - PaCO₂/RQ - PaO₂ = FiO₂ × (760 - 47) - PaCO₂/0.8 - PaO₂ = 713 × FiO₂ - PaCO₂/0.8 - PaO₂
     (A-aDO₂: Alveolar-arterial oxygen difference; FiO₂: Fraction of inspired oxygen; PB: Barometric pressure; PH₂O: Water vapor pressure; RQ: Respiratory quotient)
3. Renal Function Evaluation:
   - If Acute Kidney Injury (AKI) is confirmed, score based on creatinine value first, then multiply the score by 2. Do not multiply the creatinine value by 2 before scoring.
   - AKI defined as any of:
     - Increase in SCr by ≥0.3 mg/dl (≥26.5 μmol/l) within 48 hours;
     - Increase in SCr to ≥1.5 times baseline within the past 7 days;
     - Urine output <0.5 ml/kg/h for 6 hours.
4. Glasgow Coma Scale (GCS Score)

| **Eye Opening (E)** | **Verbal Response (V)** | **Verbal Response (V) for Intubated Patients** | **Motor Response (M)** |
| --- | --- | --- | --- |
| 4 Spontaneous | 5 Oriented | 5 Good Orientation | 6 Obeys Commands |
| 3 To Speech | 4 Confused | 3 Intermediate | 5 Localizes Pain |
| 2 To Pain | 3 Inappropriate Words | 1 No Response | 4 Withdraws |
| 1 None | 2 Incomprehensible Sounds | This column is used for the language score of patients with intubation or tracheostomy. | 3 Flexion |
|  | 1 None |  | 2 Extension |
|  |  |  | 1 None |

1. **Age Score = ( ) points**

| **Age** | **<44** | **45-54** | **55-64** | **65-74** | **>75** | **Patient Age** | **Score** |
| --- | --- | --- | --- | --- | --- | --- | --- |
| **Score** | 0 | 2 | 3 | 5 | 6 |  |  |

1. **Chronic Health Score = ( ) points**

| **If the following serious diseases or immunosuppressive states exist, the score will be performed. If it does not exist, the score for this item will be zero.** | |
| --- | --- |
| **Liver**: | Biopsy-proven cirrhosis, documented portal hypertension, prior upper GI bleed due to portal hypertension, prior hepatic failure/encephalopathy/coma |
| **Cardiovascular**: | NYHA Class IV |
| **Respiratory**: | Chronic restrictive, obstructive, or vascular diseases causing severe exercise limitation; documented chronic hypoxia, hypercapnia, secondary polycythemia, severe pulmonary hypertension (>40mmHg), or ventilator dependence |
| **Renal**: | Receiving chronic dialysis |
| **Immunosuppression**: | Immunosuppressive therapy, chemotherapy, radiotherapy, long-term or recent high-dose steroids, immunosuppressive disease (e.g., leukemia, lymphoma, AIDS) |

Presence of above conditions ○ Yes ○ No

| **Chronic Health Status** | **No Organ Failure** | **Organ Failure, Elective Surgery** | **Organ Failure, Non-surgical or Emergency Surgery** | **Patient Chronic Health Status** | **Score** |
| --- | --- | --- | --- | --- | --- |
| **Score** | 0 | 2 | 5 |  |  |

**APACHEII Score = A+B+C = ( )**

**SOFA Score**

| **Organ System** | **Parameter** | **Score Values** | | | | | **Value** | **Score** |
| --- | --- | --- | --- | --- | --- | --- | --- | --- |
|  |  | 0 | 1 | 2 | 3 | 4 |  |  |
| **Respiratory** | PaO₂/FiO₂ (mmHg) (kPa) | ≥400 (53.3) | <400 (53.3) | <300 (40) | <200 (26.7) with MV | <100 (13.3) with MV |  |  |
| **Coagulation** | Platelets (×10⁹/L) | ≥150 | <150 | <100 | <50 | <20 |  |  |
| **Liver** | Bilirubin mg/dL (μmol/L) | <1.2 (20) | 1.2-1.9 (20-32) | 2.0-5.9 (33-101) | 6.0-11.9 (102-204) | >12.0 (204) |  |  |
| **Cardiovascular** | Mean Arterial Pressure (MAP) (mmHg) or Vasopressor Requirement | MAP ≥70 | MAP <70 | Dopamine ≤5 or Dobutamine (any dose) | Dopamine >5 or Epinephrine ≤0.1 or Norepinephrine ≤0.1 | Dopamine >15 or Epinephrine >0.1 or Norepinephrine >0.1 |  |  |
| **Neurological** | Glasgow Coma Scale (GCS) | 15 | 13-14 | 10-12 | 6-9 | <6 |  |  |
| **Renal** | Creatinine mg/dL (μmol/L) | <1.2 (110) | 1.2-1.9 (110-170) | 2.0-3.4  (171-299) | 3.5-4.9  (300-440) | >5.0 (440) |  |  |
|  | Urine Output (mL/24h) |  |  |  | 201-500 | <200 |  |  |

Note: 1. Use the worst value each day. 2. Higher score indicates worse prognosis.

**SOFA Score: ( ) points**

**Microbiological Examination Record Sheet**


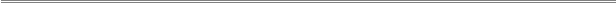


mNGS Result ○ Yes ○ No

mNGS Test Date:

Bacteria ○ No ○ Yes, please specify bacteria and sequence number

Fungi ○ No ○ Yes, please specify fungi and sequence number

Virus ○ No ○ Yes, please specify virus and sequence number

| **Bacterial Name** | \| **Sequence Number** \| \| --- \| \|  \| |
| --- | --- | --- | --- |
|  |  |
|  |  |
|  |  |
|  |  |
| **Fungal Name** |  |
|  |  |
|  |  |
|  |  |
|  |  |
| **Viral Name** |  |
|  |  |
|  |  |
|  |  |
|  |  |

| **Clinical Pathogen Test** | **Result** | **Test Date** | **Susceptibility Test** |
| --- | --- | --- | --- |
| Lower respiratory specimen smear: ○ Sputum ○ Tracheal Aspirate ○ BALF |  |  | ○ Yes ○ No |
| Lower respiratory specimen culture: ○ Sputum ○ Tracheal Aspirate ○ BALF |  |  | ○ Yes ○ No |
| Blood culture: |  |  | ○ Yes ○ No |
| Other (If none, write "None"): |  |  |  |

| **Virus** | **Specimen Type** | **Test Date** | **Nucleic Acid Test Result** | **Blood IgG Result** | **IgM Result** | **Test Date** |
| --- | --- | --- | --- | --- | --- | --- |
| Influenza A | ○ Throat swab ○ Tracheal Aspirate ○ BALF |  | ○ Negative  ○ Positive |  | ○ Negative ○ Positive |  |
|  | ○ Throat swab ○ Tracheal Aspirate ○ BALF |  | ○ Negative  ○ Positive |  | ○ Negative ○ Positive |  |
|  | ○ Throat swab ○ Tracheal Aspirate ○ BALF |  | ○ Negative  ○ Positive |  | ○ Negative ○ Positive |  |
| Influenza B | ○ Throat swab ○ Tracheal Aspirate ○ BALF |  | ○ Negative  ○ Positive |  | ○ Negative ○ Positive |  |
|  | ○ Throat swab ○ Tracheal Aspirate ○ BALF |  | ○ Negative  ○ Positive |  | ○ Negative ○ Positive |  |
|  | ○ Throat swab ○ Tracheal Aspirate ○ BALF |  | ○ Negative  ○ Positive |  | ○ Negative ○ Positive |  |
| SARS-CoV-2 | ○ Throat swab ○ Tracheal Aspirate ○ BALF |  | ○ Negative  ○ Positive |  | ○ Negative ○ Positive |  |
|  | ○ Throat swab ○ Tracheal Aspirate ○ BALF |  | ○ Negative  ○ Positive |  | ○ Negative ○ Positive |  |
|  | ○ Throat swab ○ Tracheal Aspirate ○ BALF |  | ○ Negative  ○ Positive |  | ○ Negative ○ Positive |  |
| **Other Viruses** | **Specimen** | **Test Date** | **Test Item** | **Result** |  |  |
| CMV (DNA) | ○ BALF  ○ Blood |  |  |  |  |  |
| EBV (DNA) | ○ BALF  ○ Blood |  |  |  |  |  |
|  | ○ Throat swab ○ Tracheal Aspirate ○ BALF ○ Blood |  |  |  |  |  |
|  | ○ Throat swab ○ Tracheal Aspirate ○ BALF ○ Blood |  |  |  |  |  |
|  | ○ Throat swab ○ Tracheal Aspirate ○ BALF ○ Blood |  |  |  |  |  |
|  | ○ Throat swab ○ Tracheal Aspirate ○ BALF ○ Blood |  |  |  |  |  |
|  | ○ Throat swab ○ Tracheal Aspirate ○ BALF ○ Blood |  |  |  |  |  |
|  | ○ Throat swab ○ Tracheal Aspirate ○ BALF ○ Blood |  |  |  |  |  |

**microbiological rapid on-site evaluation (mROSE)**

| **Sampling location** | | | | | | | |
| --- | --- | --- | --- | --- | --- | --- | --- |
|  | Lung |  | | Lobe |  | Segment | |
| **M-ROSE Record** | | |  | | | | |
| Date | | |  | | | | |
| Sample Type | | | ○ BALF ○ Tracheal Aspirate ○ Sputum | | | | |
| Total Lavage Volume (mL) | | |  | | | | |
| Return Volume (mL) | | |  | | | | |
| Specimen Retained for Testing | | | ○ No ○ Yes mL | | | | |
| Sample Appearance | | | ○ Viscous ○ Thin ○ Bloody ○ Turbid | | | | |
| 1. Sample Quality Control | | | ○ Excellent ○ Good ○ Poor | | | | |
| Squamous Epithelial Cell Percentage | | | % | | | | |
| Columnar Epithelial Cell Percentage | | | % | | | | |
| Red Blood Cell Percentage | | | % | | | | |
| Neutrophil Phagocytosis Phenomenon | | | ○ No ○ Yes (If yes, please specify pathogen type) | | | | |
| **Microorganism Types** | | |  | | | | |
| (1) Bacteria | | |  | | | | |
| Gram-positive Cocci | | | ○ - ○ + ○ ++ ○ +++ | | Specific species: | |  |
| Gram-negative Bacilli | | | ○ - ○ + ○ ++ ○ +++ | | Specific species: | |  |
| Gram-positive Bacilli | | | ○ - ○ + ○ ++ ○ +++ | | Specific species: | |  |
| Gram-negative Cocci | | | ○ - ○ + ○ ++ ○ +++ | | Specific species: | |  |
| (2) Fungi | | |  | | | | |
| Yeast | | | ○ - ○ + ○ ++ ○ +++ | | Specific species: | |  |
| Mold | | | ○ - ○ + ○ ++ ○ +++ | | Specific species: | |  |

**Clinical Impression:**
