# Supplementary material for: Multi-modal data to identify key factors influencing lung injury in ARDS patients undergoing invasive mechanical ventilation: A prospective multi-center observational study protocol
Source: PLoS One. 2026 Jan 23;21(1):e0332985. doi: 10.1371/journal.pone.0332985 (PMC12829816; doi:10.1371/journal.pone.0332985)

**CRF (Version Number: V1.0 Version Date: September 30, 2022)**

**Cohort Study on Factors of Lung Injury in ARDS Patients Undergoing Invasive Mechanical Ventilation**

**Case Report Form D1 -28**

Subject ID: □□-□□□-□□□□

Name Abbreviation: □□□□

Visit Date: □□□□ Year □□Month □□Day

Following Physician:

**Antibiotic Usage Record Form**


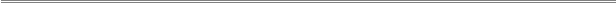


| **Antibiotic Category** | **Specific Drug Name** | **Dose & Frequency** | **Cumulative Use (Days)** | **Drug Concentration Monitoring** | **Treatment Adjustment After Monitoring** | **Drug Concentration Monitoring** | **Treatment Adjustment After Monitoring** | **Adjusted Start Date** | **Adjusted End Date** | **Total Days** |  |
| --- | --- | --- | --- | --- | --- | --- | --- | --- | --- | --- | --- |
| **β-lactam antibiotics** | | | | | | | | | | |  |
| Penicillins |  |  |  |  |  |  |  |  |  |  |  |
| 2nd gen cephalosporins (Cefuroxime, Cefotiam, Cefamandole) |  |  |  |  |  |  |  |  |  |  |  |
| 3rd gen cephalosporins (Cefotaxime, Ceftizoxime, Ceftriaxone, Cefodizime, Ceftazidime, Cefoperazone, Cefpiramide, Cefmenoxime) |  |  |  |  |  |  |  |  |  |  |  |
|  |  |  |  |  |  |  |  |  |  |  |  |
| 4th gen cephalosporins (Cefepime, Cefpirome) |  |  |  |  |  |  |  |  |  |  |  |
| Cephalosporin + enzyme inhibitor (Cefoperazone/Sulbactam, Ceftazidime/Avibactam) |  |  |  |  |  |  |  |  |  |  |  |
| Cephamycins (Cefmetazole) |  |  |  |  |  |  |  |  |  |  |  |
| Others |  |  |  |  |  |  |  |  |  |  |  |
| **Carbapenems** | | | | | | | | | | |  |
| Biapenem |  |  |  |  |  |  |  |  |  |  |  |
| Ertapenem |  |  |  |  |  |  |  |  |  |  |  |
| Imipenem/Cilastatin |  |  |  |  |  |  |  |  |  |  |  |
| Meropenem |  |  |  |  |  |  |  |  |  |  |  |
| Others |  |  |  |  |  |  |  |  |  |  |  |
| **Fluoroquinolones** | | | | | | | | | | |  |
| Levofloxacin |  |  |  |  |  |  |  |  |  |  |  |
| Moxifloxacin |  |  |  |  |  |  |  |  |  |  |  |
| Ciprofloxacin |  |  |  |  |  |  |  |  |  |  |  |
| Other quinolones |  |  |  |  |  |  |  |  |  |  |  |
| **Macrolides** | | | | | | | | | | |  |
| Azithromycin |  |  |  |  |  |  |  |  |  |  |  |
| Erythromycin |  |  |  |  |  |  |  |  |  |  |  |
| Roxithromycin |  |  |  |  |  |  |  |  |  |  |  |
| Other macrolides |  |  |  |  |  |  |  |  |  |  |  |
| **Aminoglycosides** | | | | | | | | | | |  |
| Amikacin |  |  |  |  |  |  |  |  |  |  |  |
| Others |  |  |  |  |  |  |  |  |  |  |  |
| **Anti-Gram-positive agents** | | | | | | | | | | |  |
| Vancomycin |  |  |  |  |  |  |  |  |  |  |  |
| Linezolid |  |  |  |  |  |  |  |  |  |  |  |
| Teicoplanin |  |  |  |  |  |  |  |  |  |  |  |
| Daptomycin |  |  |  |  |  |  |  |  |  |  |  |
| Others |  |  |  |  |  |  |  |  |  |  |  |
| **Tetracyclines** | | | | | | | | | | |  |
| Minocycline |  |  |  |  |  |  |  |  |  |  |  |
| Tigecycline |  |  |  |  |  |  |  |  |  |  |  |
| Doxycycline |  |  |  |  |  |  |  |  |  |  |  |
| Omadacycline |  |  |  |  |  |  |  |  |  |  |  |
| Eravacycline |  |  |  |  |  |  |  |  |  |  |  |
| Others |  |  |  |  |  |  |  |  |  |  |  |
| **Colistin** | | | | | | | | | | |  |
| Polymyxin B |  |  |  |  |  |  |  |  |  |  |  |
| Other |  |  |  |  |  |  |  |  |  |  |  |
| **Antifungals** | | | | | | | | | | |  |
| Fluconazole |  |  |  |  |  |  |  |  |  |  |  |
| Caspofungin |  |  |  |  |  |  |  |  |  |  |  |
| Voriconazole |  |  |  |  |  |  |  |  |  |  |  |
| Posaconazole |  |  |  |  |  |  |  |  |  |  |  |
| Isavuconazole |  |  |  |  |  |  |  |  |  |  |  |
| Amphotericin B |  |  |  |  |  |  |  |  |  |  |  |
| Liposomal Amphotericin B |  |  |  |  |  |  |  |  |  |  |  |
| Others |  |  |  |  |  |  |  |  |  |  |  |
| **Other Antibiotics** | | | | | | | | | | |  |
| Antibiotic Class | Drug Name | Dose/Freq. | Start Time | End Time | Cumul. Days | Regimen Adj. | Start Time |  |  |  |  |
|  |  |  |  |  |  |  |  |  |  |  |  |
|  |  |  |  |  |  |  |  |  |  |  |  |
|  |  |  |  |  |  |  |  |  |  |  |  |
| **Antiviral Medications** | | | | | | | | | | |  |
| Class | Drug Name | Dose/Freq. | Start Time | End Time | Cumul. Days | Regimen Adj. | Start Time |  |  |  |  |
|  |  |  |  |  |  |  |  |  |  |  |  |
|  |  |  |  |  |  |  |  |  |  |  |  |
|  |  |  |  |  |  |  |  |  |  |  |  |
| **Immunosuppressants** | | | | | | | | | | |  |
| Class | Drug Name | Dose/Freq. | Start Time | End Time | Cumul. Days | Regimen Adj. | Start Time |  |  |  |  |
|  |  |  |  |  |  |  |  |  |  |  |  |
|  |  |  |  |  |  |  |  |  |  |  |  |
|  |  |  |  |  |  |  |  |  |  |  |  |
|  |  |  |  |  |  |  |  |  |  |  |  |

**Vasoactive Agents and Sedation/Analgesia Usage**


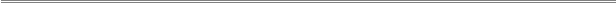


| **Use of vasoactive agents: ○ Yes ○ No** | | |
| --- | --- | --- |
| **Drug** | **Dose/Concentration/Speed** | **Notes (μg/kg*min)** |
| Dopamine |  |  |
| Dobutamine |  |  |
| Norepinephrine |  |  |
| Metaraminol |  |  |
| Other vasoactive agents |  |  |
| **Use of sedatives/analgesics: ○ Yes ○ No** | | |
| **Drug** | **Dose/Concentration/Speed** | **Notes (μg/kg*min)** |
| Fentanyl |  |  |
| Remifentanil |  |  |
| Propofol |  |  |
| Midazolam |  |  |
| Dexmedetomidine |  |  |
| Other sedatives/analgesics |  |  |
| **Use of neuromuscular blockers: ○ Yes ○ No** | | |
| **Drug** | **Dose/Concentration/Speed** | **Notes (μg/kg*min)** |
| Rocuronium |  |  |
| Atracurium |  |  |
| Others |  |  |

**Adverse Event Report Form**


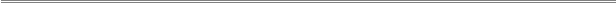


**Did any adverse events occur during the study?**
○ No
○ Yes → Please complete the table below

**Adverse Event Definition:**
*Any untoward medical occurrence in a subject who has signed the informed consent form and been enrolled in a clinical trial, from the time of enrollment through the final follow-up visit, regardless of whether it has a causal relationship with the study.*

| **Adverse Event Name** | **Start Date (YYYY/MM/DD)** | **Severity** | **Management Measures** | **Relationship to Intervention** | **Outcome** | **Recovery Date Confirmed?** | **Recovery Date (YYYY/MM/DD)** | **SAE?** | **Withdrawn due to AE?** |
| --- | --- | --- | --- | --- | --- | --- | --- | --- | --- |
|  |  |  |  |  |  |  |  |  |  |
|  |  |  |  |  |  |  |  |  |  |
|  |  |  |  |  |  |  |  |  |  |

**New Systemic Illnesses During Hospitalization**


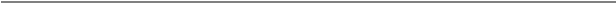


Date of clinical change during hospitalization:

Cerebral infarction

Myocardial infarction

Arrhythmia

Gastrointestinal bleeding

Liver dysfunction

Other: __________


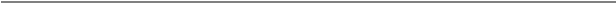


**Clinical Outcome Observation Form**


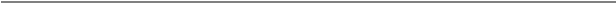


| **Discharged from ICU:** | □No □Yes Date: ________ |
| --- | --- |
| Extubation? | □No □Yes Date: ________ |
| ICU outcome (≤28 days) | □Survived □Died Date: ________ |
| 28-day outcome | □Survived □Died Date: ________ |
| CRRT | □No □Yes Total CRRT duration (hours): ________ |

**Early Termination Form**


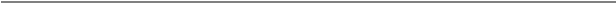


Date: ________
Primary reason for early termination (select one):
□ Adverse event making continuation unsuitable (Complete AE form)
□ Subject withdrawal request
□ Protocol violation (Specify): ____________________
□ Lost to follow-up
□ Other (Specify): ____________________
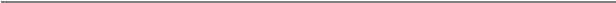


**Study Completion Form**
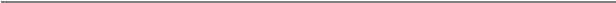


Did subject complete study? □Yes □No
Completion date: ________
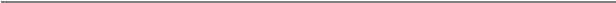


**CRF Review Statement**


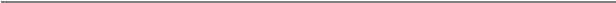


*I confirm that all entries in this CRF are accurate, complete, and verifiable.*
Investigator Signature: ____________________

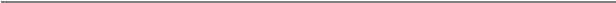

Supplement: S4 File — Case report form for days 1–28, excluding day 1 and day 3. (DOCX) [file pone.0332985.s004.docx]
