# Supplementary material for: Multi-modal data to identify key factors influencing lung injury in ARDS patients undergoing invasive mechanical ventilation: A prospective multi-center observational study protocol
Source: PLoS One. 2026 Jan 23;21(1):e0332985. doi: 10.1371/journal.pone.0332985 (PMC12829816; doi:10.1371/journal.pone.0332985)
Supplement: S5 File — Methodological description of the multi-omics detection approaches to be used. (DOCX) [file pone.0332985.s005.docx]

**mNGS pathogen diagnosis, metagenomic and metatranscritomic sequencing**

Immediately after collection, undiluted bronchoalveolar lavage fluid (BALF) samples will be transported to Vision Medicals Co., Ltd. for metagenomic next-generation sequencing (mNGS)-based pathogen diagnosis, metagenomic microecology detection, and viromic detection: mNGS pathogen diagnosis (IDseq™ Ultra) will be initiated promptly upon sample arrival, with nucleic acid extraction and library construction performed using kits VMRS0107-50 and VMRS0108-50, followed by sequencing on the Visionseq 1000 platform with a single-end 75 bp strategy. The target sequencing depth for each sample is 20 million reads, and the actual sequencing depth must be no less than 10 million reads. After sequencing, raw reads will be processed via Trimmomatic (v0.39) to remove adapter contamination, low-quality reads, duplicates, and sequences < 40 bp, with low-complexity reads further filtered using fastp (v0.23.4) under default settings, filtered reads aligned to the human GRCh38 reference genome via Burrows-Wheeler Aligner (BWA-0.7.17) to identify and exclude human-derived sequences, taxonomic classification of microbial reads conducted using Kraken2 (v2.1.3) with default parameters (confidence = 0.2, threads = 40), microbial reads re-aligned to the same reference database via BWA (v0.7.17) to reduce false-positive alignments from PCR duplicates or low-complexity regions (retaining only alignments with ≥96% nucleotide identity and excluding reads mapping to multiple loci within the same genus to avoid taxonomic ambiguity), and read counts normalized using the reads per million (RPM) method to correct for inter-sample sequencing depth differences. Metagenomic microbial analyis and metatranscriptomic viral analyses will be conducted after all samples are collected to mitigate batch effects, with sequencing performed on the DNBSEQ-T7 platform (Novizan) using a paired-end 150 bp strategy (minimum 10 Gb per sample, Phred quality score Q30 ≥ 85%), all samples in each batch processed strictly per standardized standard operating procedures (SOPs) to minimize technical variations from reagent inconsistencies and operator-dependent factors, and taxonomic classification of sequencing reads implemented via Kraken2 (v2.1.3) with default parameters (confidence = 0.2, threads = 40).

**Transcriptomics Analysis**

Transcriptomic sequencing will be performed using the Illumina NovaSeq X Plus platform (Illumina, USA) with paired-end 150 bp sequencing. Prior to sequencing, library quality control and quantification will be implemented as follows: After library construction, initial quantification will be conducted using a Qubit 2.0 Fluorometer, and libraries will be diluted to 1.5 ng/μL. Subsequently, an Agilent 2100 Bioanalyzer will be used to verify the insert size; upon confirmation that the insert size meets the expected criteria, quantitative real-time PCR (qRT-PCR) will be employed for accurate quantification of the effective library concentration, which is required to be ≥ 1.5 nM to ensure library quality. Library construction will utilize the Fast RNA-seq Lib Prep Kit V2 (Cat. No.: RK20306; ABclonal, China). The sequencing strategy will adopt PE150 paired-end sequencing with a sequencing depth of 2–3×. The GRCh38 genome assembly (annotation version: GRCh38.84) will serve as the reference for read alignment and gene annotation. Quality control (QC) criteria will be defined as follows: Phred quality scores Q20 > 90%, Q30 > 85%, and GC content ranging from 40% to 60%. The bioinformatics analysis workflow will proceed sequentially: First, raw reads containing adapters, missing bases, or low-quality sequences will be filtered using Fastp to obtain clean reads. Clean reads will then be aligned to the human reference genome (GRCh38) using Hisat2, with gene annotation based on the GRCh38.84 version. Transcript assembly will be performed using StringTie 2.1.5, and standardized transcript expression abundances (FPKM; Fragments Per Kilobase of exon model per Million mapped fragments) will be calculated. GffCompare will be used to evaluate the quality of transcript assembly. Following assembly, raw read counts and FPKM values for all genes will be extracted using Python scripts integrated with StringTie. Differential expression analysis will be conducted using DESeq2; genes with an adjusted P-value (padj) < 0.05 and |log2FoldChange| ≥ 1 will be defined as differentially expressed genes (DEGs). Principal component analysis (PCA) and heatmap visualization will be generated using the R packages ggplot2 and pheatmap, respectively. Functional enrichment analysis of DEGs will be performed using the R package clusterProfiler for Kyoto Encyclopedia of Genes and Genomes (KEGG) pathway enrichment, with a significance threshold of P < 0.05 after Benjamini correction. To mitigate batch effects, all samples within each batch will be processed strictly in accordance with standardized standard operating procedures (SOPs) to minimize technical variations caused by reagent inconsistencies and operator-dependent errors.

**Single-Cell RNA Sequencing**

Single-cell RNA sequencing (scRNA-seq) will be performed using the DNBSEQ-T7RS platform. Each sample will contain 20,000–30,000 cells, with cell counting conducted using a Luna Cell Counter. Library construction will employ the DNBelab C Series High-Throughput Single-Cell RNA Library Preparation Kit V3.0 (covering droplet generation, cDNA synthesis, and oligo library construction; Cat. No.: 940-001818-000). Sequencing depth requirements are as follows: 500 million reads for the cDNA library and 50 million reads for the oligo library. Paired-end sequencing will be used, with read lengths specified as 30 bp for Read Ⅰ (R1) and 100 bp for Read Ⅱ (R2) for both libraries. The hg38 genome assembly and corresponding annotations will serve as the reference for alignment and analysis.Quality control (QC) will be implemented at multiple key stages: 1. Sample Preparation QC: Cell viability > 80% and cell size < 40 μm; 2. cDNA Intermediate Amplification QC: cDNA concentration ≥ 10 ng/μL (quantified by Qubit) and fragment size ranging from 600–2000 bp (verified by Qsep); 3. cDNA Library QC: Library concentration ≥ 10 ng/μL (Qubit) and fragment distribution of 180 ± 10 bp (Qsep); 4. Oligo Library QC: Library concentration ≥ 10 ng/μL (Qubit) and fragment size ranging from 350–550 bp (Qsep).

Bioinformatics analysis will follow the single-cell transcriptome pipeline of DNBC4tools. Raw sequencing data from the cDNA and oligo libraries of each sample will undergo quality control, alignment, and functional region annotation using default parameters. Subsequently, beads will be merged, and cells will be identified to generate a raw gene expression matrix, which will then be filtered to obtain a cleaned gene expression matrix. Further analyses will include cell filtering, dimensionality reduction, clustering, and cell type annotation. Finally, an interactive report in HTML format will be generated, and the analysis results will be exported. Batch effects will be mitigated using the Harmony algorithm. The principle of this method involves applying principal component analysis (PCA) to embed transcriptomic expression profiles into a low-dimensional space, followed by an iterative process to eliminate batch-specific variations, thereby achieving robust data integration across different batches.

**Proteomics Detection and Analysis**

Proteomics analysis will be performed using a hyphenated technique combining a nano-flow high-performance liquid chromatography (HPLC) system (EASY-nLC 1200) with either an HFX mass spectrometer or an OE480 mass spectrometer. The sample processing workflow will be as follows: an appropriate amount of sample will be taken and subjected to high-abundance protein depletion using High-Select™ Top14 Abundant Protein Depletion Mini Spin Columns, followed by enzymatic digestion with trypsin to obtain peptides. After lyophilization, the peptides will be reconstituted in 100 μL of 0.1% formic acid solution, and 2% of the reconstituted sample will be injected for analysis.

Mass spectrometry will be conducted in data-independent acquisition (DIA) mode, with the parent ion scan range set to 300–1400 m/z and the full scan resolution at 60,000 at 200 m/z. Thirty DIA scans will be acquired after each full scan, and high-energy collision dissociation (HCD) will be used as the fragmentation method.

Database searching and data analysis will be performed based on a self-built spectral library and the iProteome one-stop data analysis cloud platform. Functional annotation will refer to the GO, KEGG, Pfam, Reactome, and WikiPathways databases; protein-protein interaction network analysis will be conducted using the STRING database; and gene set enrichment analysis (GSEA) will be performed with the MSigDB database.

Quality control criteria will include: the mass deviation of identified peptides will be mainly distributed within 10 ppm, and the peptide false discovery rate (Peptide FDR) will be set to ≤ 0.05.

Bioinformatics analysis will include protein clustering analysis, GO functional annotation, KEGG pathway annotation, enrichment analysis based on hypergeometric distribution test, protein-protein interaction network analysis, Reactome pathway analysis, WikiPathways analysis, and GSEA. To mitigate batch effects, a hierarchical normalization strategy will be adopted: z-score normalization will be applied to quantitative data during clustering analysis, and inter-sample normalization correction (Fraction of Total, FOT) will be used for abundance range presentation.

**Metabolomics Detection and Analysis**

Metabolomics analysis will be performed using an ACQUITY 2D ultra-performance liquid chromatography (UPLC) system (Waters) coupled with a Q Exactive (QE) high-resolution mass spectrometer (Thermo Fisher Scientific). Sample preparation will proceed as follows: Methanol will be added to each sample at a 1:4 volume ratio (sample:methanol) for metabolite extraction. Following centrifugation and supernatant collection, four 100 µL aliquots will be withdrawn for subsequent UPLC-MS/MS analysis.

To maximize metabolite coverage, four complementary UPLC-MS/MS analytical methods will be employed: (1) positive electrospray ionization (ESI+) mode with a C18 chromatographic column; (2) negative electrospray ionization (ESI−) mode with a C18 chromatographic column; (3) positive electrospray ionization (ESI+) mode with a C18 chromatographic column; (4) negative electrospray ionization (ESI−) mode with a HILIC chromatographic column. Mass spectrometry parameters will be configured as follows: scan resolution of 35,000 and a mass-to-charge ratio (m/z) scan range of 70–1000.

Metabolite identification will be accomplished by matching against a self-constructed metabolite database derived from authentic standards, adhering to strict criteria: (1) primary accurate mass variation ≤ 10 ppm; (2) consistent retention index (RI); (3) congruence between the observed primary accurate mass and secondary fragment spectra. Quality control (QC) measures will be implemented to ensure system stability: QC samples will be inserted at regular intervals throughout the entire injection sequence, with the median relative standard deviation (RSD) of internal standard signals in QC samples required to be < 5%.

Data analysis will be conducted sequentially. First, chromatographic peak extraction will be performed using a self-developed data processing system. Raw peak area data will undergo preprocessing, including log2 transformation, median normalization, and imputation of missing values with the minimum value across all samples, to minimize technical variability. Statistical analyses will be carried out using R software, encompassing parametric tests (t-test) or non-parametric tests (Wilcoxon rank-sum test) for univariate comparisons. Multivariate analyses, including principal component analysis (PCA), partial least squares discriminant analysis (PLS-DA)/orthogonal partial least squares discriminant analysis (OPLS-DA), and random forest (RF) classification, will be utilized to identify discriminative metabolites. Pathway enrichment analysis will be performed based on the KEGG database using the MetPA toolkit combined with hypergeometric testing, aiming to annotate the biological functions of differentially abundant metabolites.

Batch effects will be mitigated through a three-pronged strategy: (1) All samples will be analyzed in a single batch following completion of sample collection to eliminate inter-batch variability; (2) systematic insertion of QC samples to monitor and ensure instrumental stability throughout data acquisition; (3) standardized data preprocessing (log2 transformation, median normalization, and missing value imputation) to correct for technical biases associated with batch variation.
